# Structure-Based Function and Regulation of NCX Variants: Updates and Challenges

**DOI:** 10.3390/ijms24010061

**Published:** 2022-12-21

**Authors:** Daniel Khananshvili

**Affiliations:** Department of Physiology and Pharmacology, Sackler Faculty of Medicine, Medical School, Tel-Aviv University, Tel-Aviv 69978, Israel; dhanan@tauex.tau.ac.il; Tel.: +972-03-640-9961

**Keywords:** sodium-calcium exchange, NCX, NCKX, NCLX, CAX, Ca^2+^/CA, antiporter, ion transport mechanisms, allosteric regulation, ion selectivity, ion binding sites, transport rates, regulatory domains, post-translational modification

## Abstract

The plasma-membrane homeostasis Na^+^/Ca^2+^ exchangers (NCXs) mediate Ca^2+^ extrusion/entry to dynamically shape Ca^2+^ signaling/in biological systems ranging from bacteria to humans. The NCX gene orthologs, isoforms, and their splice variants are expressed in a tissue-specific manner and exhibit nearly 10^4^-fold differences in the transport rates and regulatory specificities to match the cell-specific requirements. Selective pharmacological targeting of NCX variants could benefit many clinical applications, although this intervention remains challenging, mainly because a full-size structure of eukaryotic NCX is unavailable. The crystal structure of the archaeal NCX_Mj, in conjunction with biophysical, computational, and functional analyses, provided a breakthrough in resolving the ion transport mechanisms. However, NCX_Mj (whose size is nearly three times smaller than that of mammalian NCXs) cannot serve as a structure-dynamic model for imitating high transport rates and regulatory modules possessed by eukaryotic NCXs. The crystal structures of isolated regulatory domains (obtained from eukaryotic NCXs) and their biophysical analyses by SAXS, NMR, FRET, and HDX-MS approaches revealed structure-based variances of regulatory modules. Despite these achievements, it remains unclear how multi-domain interactions can decode and integrate diverse allosteric signals, thereby yielding distinct regulatory outcomes in a given ortholog/isoform/splice variant. This article summarizes the relevant issues from the perspective of future developments.

## 1. Introduction

The plasma membrane Na^+^/Ca^2+^ exchangers (NCX) represent a large family of proteins that mediate Ca^2+^ entry/exit and, thus, modulate the Ca^2+^ signaling/homeostasis in biological systems ranging from bacteria to humans [1,2,3]. The mammalian NCX variants are expressed in a tissue-specific manner to fulfill cell-specific demands [3,4,5]. Thus, structurally predefined NCX variants modulate diverse physiological functions on the cellular, systemic, and organ levels [6,7,8,9,10,11,12]. Altered expression/regulation of NCXs is associated with many maladies, although the underlying molecular and cellular mechanisms remain unresolved [9,10,11,12,13,14,15]. Selective pharmacological targeting of tissue-specific NCX variants could benefit many clinical applications [16,17,18,19,20,21,22,23]. However, the structure-based pharmacological targeting remains unrealized mainly because the underlying molecular mechanisms of NCX [24,25,26,27,28,29] and other antiporters [30,31,32,33] remain incompletely resolved. 

Although NCX orthologs, isoforms, and their splice variants share some common structural motifs, they exhibit striking differences in the ion-transport rates and regulatory modes [24,25,26]. To date, the full-size crystal structures of the archaeal NCX (NCX_Mj) [27,28] and prokaryotic CAXs [34,35,36] are available, whereas protein folding details of any eukaryotic NCX remains undiscovered. The structural resolution of isolated regulatory domains, derived from mammalian and invertebrate NCXs, in combination with functional and biophysical studies [37,38,39,40,41,42] provided meaningful clues on the molecular mechanisms underlying the regulatory variances among eukaryotic NCXs. Despite this progress, the currently available structural information is insufficient to resolve remote allosteric interactions between the regulatory and ion-transporting domains in eukaryotic NCXs. Therefore, the challenge is to elucidate structure-based functional and regulatory mechanisms that can rationally explain how the tissue-specific NCX variants can diversify, transfer and integrate multifaceted allosteric signals. 

Over the last decade, significant progress has been made in understanding the structure-dynamic mechanisms underlying ion transport and regulation in NCXs and similar proteins by exploring X-ray crystallography, molecular dynamics (MD) simulations, HDX-MS, NMR, SAXS, and extended analyses of mutational effects on the ion fluxes. This article summarizes the structure-dynamic mechanisms underlying ion selectivity, transport rates, and regulation in NCX variants. 

## 2. Molecular Hallmarks of Prokaryotic and Eukaryotic NCXs

Even though it is widely accepted that the phenomenological rules of ion transport are fundamentally different in channels and carrier-type secondary transporters (such as NCXs) and pumps, the structure-dynamic details underlying the ion transport mechanisms remain incompletely understood [30,31,32,33]. The crystal structures of the archaeal NCX (NCX_Mj) [26,27], prokaryotic CAXs [34,35,36], and the isolated regulatory domains (CBD1 and CBD2) of eukaryotic NCXs [37,38,39,40,41,42] have provided new opportunities for investigating the unifying mechanisms underlying ion transport and regulation in NCX and similar proteins [3,8,24,25,26]. Despite this progress, some fundamental issues remain unresolved—e.g., it is unclear what determines the kinetic and regulatory variances among NCX variants [3,4,5,6,7,8,24,25]. The future discovery of eukaryotic NCX structure (using Cryo-EM) may provide breakthrough information on cell-specific responses of NCX variants to Ca^2+^ signaling/homeostasis. This progress may allow selective pharmacological targeting of tissue-specific NCX variants, which has a huge biomedical potential. 

### 2.1. NCX Proteins Share Some Basic Structural Motifs with Ca^2+^/CA Proteins

The superfamily of the Ca^2+^/CA (Ca^2+^/Cation) exchangers (antiporters) contains several gene families (NCX, NCKX, NCLX, CCX, and CAX) whose members can translocate Ca^2+^ across the membrane in exchange for Na^+^, K^+^, H^+^, Li^+^, or Mg^2+^ [3,5,43,44,45,46,47,48]. These variances in ion selectivity of counter ion are of primary physiological significance since this permits the utilization of Na^+^, K^+^, and/or H^+^ gradients in a given cell type [44,45,46,47]. Although the protein sequence alignments of prokaryotic and eukaryotic Ca^2+^/CAs show striking similarities, only the crystal structures of prokaryotic NCX_Mj and CAX proteins are currently available [26,27,34,35,36]. The structural details of eukaryotic Ca^2+^/CA proteins remain challenging to discover even using the most advanced techniques, like Cryo-EM. 

**Figure 1:**
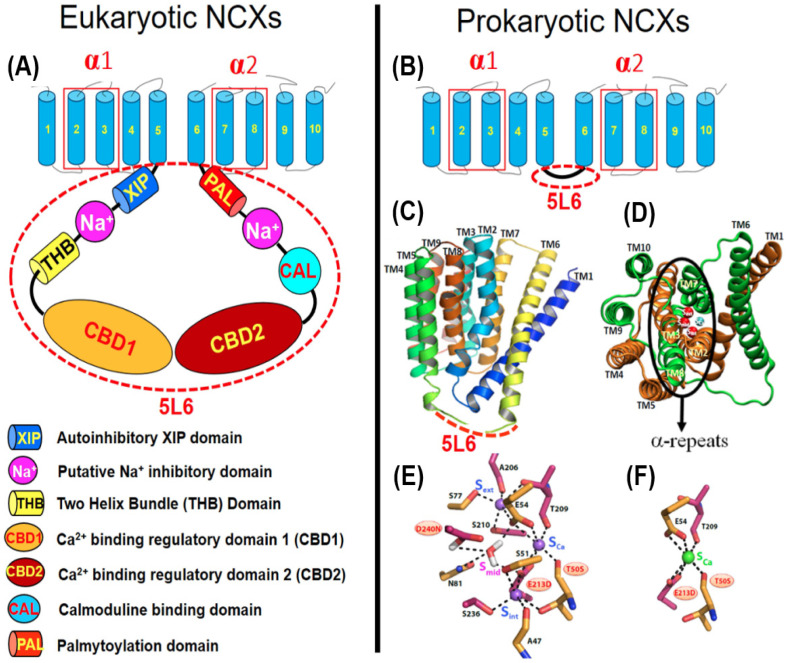
Structure-topological arrangements of prokaryotic and eukaryotic NCX proteins. Eukaryotic (**A**) and prokaryotic (**B**) NCX proteins consist of 10 trans-membrane helices (TM1–10), where highly conserved α-repeat regions form a four-helix ion passageway with TM2/TM3 (α_1_) and TM7/TM8 (α_2_) bands (shown boxed). Eukaryotic NCXs contain a very long (~520 residues) cytosolic 5L6 loop (f-loop) between TM5 and TM6 with the Ca^2+^ binding regulatory domains, CBD1 and CBD2. In the lack of regulatory domains, the 5L6 loop of prokaryotic NCXs is very short (12–16 residues). (**C**) The crystal structure of the archaeal NCX_Mj protein (PDB 3V5U) in the OF orientation describes a tightly packed core of eight helices (TM2-TM5 and TM7–TM10), where two long and loosely packed helices (TM1 and TM6) form a two-helix slanted bundle (TM1/TM6) in front of a rigid eight-helix hub (TM2–TM5 and TM7–TM10). (**D**) The α_1_ and α_2_ repeats, covering the ion-transporting four-helix structure (TM2/TM3 and TM7/TM8), are shown from the extracellular side of the NCX_Mj (PDB 3V5U) crystal structure. The combined data, attained from X-ray crystallography, MD simulations, and mutational effects on ion fluxes, support a model according to which 3Na^+^ occupy S_ext_, S_Ca_, and S_int_ (**E**) and 1Ca^2+^ occupies S_Ca_ (**F**). The Na^+^ (PDB 5HXE) and Ca^2+^ (PDB 3V5U) coordinating residues are presented as sticks, where purple and green spheres represent the Na^+^ and Ca^2+^ ions, respectively. The S_mid_ site is occupied by a water molecule (the oxygen and hydrogen atoms are donated in red and white sticks, respectively).

In similarity with other Ca^2+^/CAs, the prokaryotic and eukaryotic NCXs contain ten transmembrane helices (TM1–TM10), where two hubs (TM1–TM5 and TM6–TM10) are inversely oriented to form an inverted twofold pseudo-symmetry [49,50] (Figure 1A,B). Most importantly, the Ca^2+^/CA proteins contain highly conserved repeats (α_1_ and α_2_) that form a four-helix entity (TM2, TM3, TM7, and TM8) for an ion passageway with four binding sites (S_ext_, S_int_, S_mid_, and S_Ca_) (Figure 1C–F). The cytosolic f-loop (5L6) connects inversely oriented hubs (TM1–TM5 and TM6–TM10) through a link between TM5 and TM6. Prokaryotic NCXs have a very short 5L6 loop (12–32 residues) due to the lack of regulatory domains, whereas the mammalian NCXs contain a huge 5L6 loop (~520 residues) with many regulatory sites (Figure 1A,B). Thus, regulatory domains account for big differences in the molecular weight between mammalian (930–970 aa) and prokaryotic (300–400 aa) NCXs (Figure 1A,B).

### 2.2. NCXs Share Ion-Exchange Stoichiometry While Owing Very Different Transport Rates

The prokaryotic and eukaryotic prototypes of the cell-membrane NCXs have an electrogenic stoichiometry (3Na^+^:1Ca^2+^) of ion exchange [51,52,53], whereas the transmembrane translocation of 1Ca^2+^- or 3Na^+^-bound species occurs in separate steps of the transport cycle [54,55,56]. The Na^+^/Ca^2+^ exchange can occur either in the forward (Ca^2+^-extrusion) or reverse (Ca^2+^-entry) mode; however, the directionality of the ion fluxes is controlled by the stoichiometry of ion transport, membrane polarization/depolarization, and the transmembrane gradients of the Na^+^ and Ca^2+^ ions [2,3,4,29,51,52,53]. Notably, the directionality of net Ca^2+^ fluxes through the NCX may change due to varying membrane potentials and ionic conditions under normal or altered physiological conditions [8,12,14,26,57]. Even though the prokaryotic and eukaryotic NCXs share a common stoichiometry of ion exchange, 3Na^+^:1Ca^2+^ [51,52,53], the mammalian NCXs mediate faster transport rates than the NCX_Mj [58,59,60]. Namely, the cardiac NCX1.1-mediated ion currents, measured using the patch-clamp techniques [58,60], and NCX_Mj-mediated ion fluxes, measured using the ^45^Ca^2+^-uptake assays [60], underscore the kinetic differences between NCX1.1 (~5000 s^−1^) and NCX_Mj (~0.5 s^−1^). Notably, the ion transport turnover rates of the archaeal and eukaryotic NCXs were not measured using the same techniques side-by-side. Nevertheless, a comparison of the Na^+^-dependent ^45^Ca^2+^-uptake data, obtained using the vesicular preparations of the cardiac NCX1.1 or NCX_Mj, revealed ~10^4^-fold differences between the two proteins in the turnover rates of the transport cycle [59,60,61,62]. More specifically, the isolated cardiac sarcolemma vesicles have much higher ^45^Ca^2+^ uptake rates (mole ^45^Ca^2+^/mg membrane protein/time unit) than the vesicles containing NCX_Mj, even though the site density of NCX_Mj (~10% of total membrane protein) is incomparably higher than of those NCX1.1 (<0.1% of total membrane protein) [60,61,62]. It remains unclear whether the other prokaryotic NCXs also own low transport rates or NCX_Mj is an exemption. Side-by-side experiments, using the same methods of ion-flux assays, are required to compare the kinetic capacities of mammalian and prokaryotic NCXs. Notably, the regulatory domains of eukaryotic NCXs cannot be the reason for differences in kinetics since the proteolytic shaving or the genetic deletion of regulatory domains results in maximal ion currents, mediated by NCX1.1 [63].

### 2.3. NCX Can Mediate Either the Ca^2+^-Exit (Forward) or Ca^2+^-Entry (Reverse) Mode

The NCX-mediated ion exchange can occur either in the forward (Ca^2+^-exit) or reverse (Ca^2+^-entry) mode [12,57]. The directionality of the Ca^2+^ movements depends on the [Na^+^]_o_, [Na^+^]_I_, [Ca^2+^]_o_, [Ca^2+^]_i_, and the membrane potential charge [2,8,12,14,57]. At typical values of [Na^+^]_o_, [Na^+^]_i_, [Ca^2+^]_o_, and [Ca^2+^]_i_ (under resting conditions), the electrochemical null point, or reversal potential (E_NCX_), for NCX would be in the range of ~−30 mV, and the directionality of net Ca^2+^ flux. The directionality of net Ca^2+^ fluxes depends on the membrane potential (E_m_) and E_NCX_ (including ion concentrations and stoichiometry of ion exchange) [12,14,26,57]. Namely, whenever E_m_ > E_NCX_, the Ca^2+^ entry through NCX takes place, and when E_m_ < E_NCX_, the extrusion is preferred. In excitable tissues (e.g., ventricle myocytes or neurons) the dynamic swings in the [Na^+^]_i_, [Ca^2+^]_i_, and membrane potential during the action potential influence the directionality of the net Ca^2+^ movements through NCX, since E_m_ alternates between −90 mV and +50 mV) [2,8,57]. Since the NCX-mediated ion exchange is electrogenic, membrane depolarization and increases in [Na^+^]_i_ foster the reverse mode of NCX, whereas hyperpolarization or elevated [Ca^2+^]_i_ favor the forward mode of NCX. Thus, NCXs’ directionality and transport rates are controlled by transmembrane gradients of Na^+^ and Ca^2+^, ion-exchange stoichiometry, and membrane potential. The effects of these factors become very dynamic during the action potential, where the directionality of charge/ion fluxes reverses and ion-exchange rates permute (up to 50-fold) within a few milliseconds [2,14,26,57]. Thus, from one side, the electrochemical gradient tightly controls the kinetics and directionality of NCX function, whereas any dynamic changes in the NCX activity (due to the allosteric regulation) can dynamically feedback the cytosolic Na^+^ and Ca^2+^ levels in a given cell type. Thus, this dynamic loop between NCX and ion signaling/homeostasis contains both the kinetic and thermodynamic elements, which are cell-specific, in nature.

Although the Ca^2+^ exit (the forward mode) represents a major physiological mode of NCX operation in most cell types, the Ca^2+^ entry mode plays a critical role in some cell types (e.g., in glial and epithelial cells). For example, the Ca^2+^ entry occurs in some epithelial cells, where the resting membrane potential (E_m_) approaches the reversal potential of NCX (E_NCX_ ≈ −30 mV). Moreover, even small changes in the basal values of E_m_ and/or E_NCX_ can reverse the ion-exchange directionality. Notably, even twofold changes in cytosolic [Na^+^]_i_ can dramatically affect E_NCX_, since any changes in [Na]_i_ are powered in the third degree due to the 3Na^+^:1Ca^2+^ stoichiometry [2,8,26,57]. For example, a transient increase in cytosolic Na^+^, induced by glutamate or GABA uptake into astrocytes, can mediate Ca^2+^ entry through NCX [12,14]. This Na^+^-dependent reversal of the Ca^2+^ exit/entry modes couples the Na^+^ and Ca^2+^ signaling in glial cells to couple neuron-glia interactions; this might play a critical role when Na^+^ and/or Ca^2+^ overload occurs under pathophysiological conditions [8,12,14]. Notably, the reverse mode of NCX (Ca^2+^ entry) may significantly contribute to the cellular and systemic functions in some species (e.g., lobster and squid giant axons among others) while supporting the physiologically relevant Ca^2+^ uptake mechanism under given ionic concentrations [2,29,64,65,66]. The Ca^2+^ entry through NCX becomes a favorable mode in water-living species under conditions in which extracellular Na^+^ concentrations are relatively low versus high concentrations of extracellular Ca^2+^ [2,29]. The crustacean model of the NCX-mediated Ca^2+^-entry seems very similar to the Ca^2+^-entry mechanism found in fish gills, except that the apical carrier-mediated Ca^2+^ uptake in fish has been challenging to demonstrate [64,65,66].

### 2.4. Bidirectional Ion Access/Transport Is Asymmetric in NCX_Mj and Eukaryotic NCX

Biochemical, biophysical, and kinetic analyses provided complementary data revealing that the ion access/translocation at the extracellular and cytosolic vestibules is asymmetric either in eukaryotic or prokaryotic NCX orthologs [3,8,26,60,63,67,68,69]. Despite the huge differences in the transport rates, NCX_Mj and NCX1.1 exhibit a comparable degree of asymmetry in bidirectional ion transport while showing 10–50-fold differences between the K_m_^Cyt^ and K_m_^Ext^ values for either Na^+^ or Ca^2+^ [2,3,6,26,60]. In agreement with this, the ATR-FTIR and 2D IR analyses revealed that the high and low-affinity values of Na^+^ binding (K_d_) to purified NCX_Mj are comparable with the K_m_^Cyt^ and K_m_^Ext^ values obtained from ion-flux assays [70,71]. Moreover, mutational analysis of ion-passageway residues and HDX-MS analysis revealed that specific structural elements are associated with the functional asymmetry of NCX_Mj [60,68,69,72]. The lopsided affinities for ion interactions with NCX (reflecting the functional asymmetry for ion access/transport at the opposite sides of the membrane) may represent an evolutionary adaptation of NCX proteins to given concentrations of ions under physiological conditions. The ionic concentrations at the cytosolic and extracellular sides are asymmetric in most physiological systems. However, in some invertebrate organisms, the cytosolic and extracellular K_m_ values of Na^+^ appear to be comparable due to relatively low concentrations of extracellular Na^+^ [2,29]. Notably, during the evolution, the inversely oriented repetitive structures (such as α_1_ and α_2_) have been generated through gene duplication and fusion to produce functionally diverged enzymes, channels, receptors, and transporters [49,50,72].

### 2.5. Structure-Based Divergence of NCX Regulation Is Critical for Cell-Specific Functions

**Figure 2:**
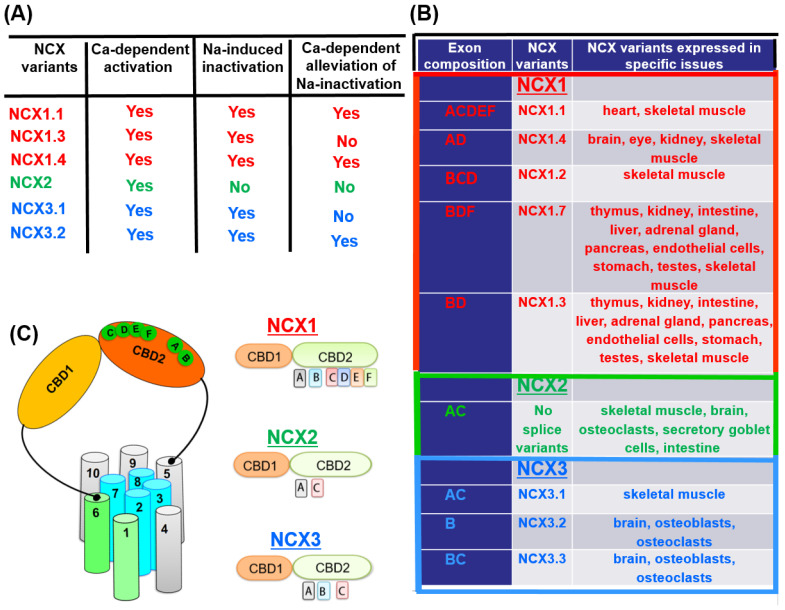
Isoform/splice variants of eukaryotic NCX variants. (**A**) The eukaryotic NCX variants share a mode of Ca^2+^-dependent activation (due to the Ca^2+^ binding to CBD1). NCX1 and NCX3 display a mode of Na^+^-induced inactivation (due to the Na^+^ binding to some unknown site). NCX2 lacks the Na^+^-induced inactivation mode. The Ca^2+^ binding to CBD2 can alleviate the Na^+^-induced inactivation only in the splice variants of NCX1 and NCX3 that have the Ca^2+^ binding sites at CBD2. Exon A generates the Ca^2+^ binding sites in CBD2 of NCX1 (but not of NCX3). In contrast, exon B breads the Ca^2+^ sites in CBD2 of NCX3 (but not of NCX1). (**B**) The protein products of NCX1, NCX2, and NCX3 genes (isoforms) are expressed in a tissue-specific manner, thereby underscoring diverse regulatory responses of NCX variants to cell-specific Ca^2+^ signaling/homeostasis. (**C**) The Ca^2+^-binding regulatory domains, CBD1 and CBD2, form a head-to-tail tandem of two regulatory domains (CBD12). The alternatively spliced region is located exclusively with CBD2. The splice variants of NCX1 and NCX3 arise from a combination of six small exons (A, B, C, D, E and F), where a mutually exclusive exon (either A or B) appears in every splice variant in different combinations of the cassette exons (C, D, E, F). NCX2 contains exons A and C, although no splice variants were found for NCX2.

Even though the mammalian NCX proteins are low abundant proteins (<10^6^ copies per cell), they chiefly contribute to Ca^2+^ signaling/homeostasis since mammalian NCXs mediate high transport rates for Ca^2+^ entry/exit to fulfill cell-specific functional requirements [1,2,3,6,55,56,57,58,59]. The much lower transport rates (see above), owned by the archaeal NCX_Mj [60], underscore rather slower dynamics of Ca^2+^ signaling in prokaryotic cells, even though prokaryotic NCX_Mj and eukaryotic NCXs share a common stoichiometry of ion exchange, 3Na^+^:1Ca^2+^ [51,52,53]. Moreover, mammalian NCX isoform/splice variants have distinct regulatory features since structurally diverged NCX variants must handle appropriate responses to cell-specific Ca^2+^ and Na^+^ signaling [12,16,31,32,57]. Therefore, mammalian gene isoforms (NCX1, NCX2, and NCX3) and their splice variants (Figure 2A,B) are expressed in a tissue-specific manner while sharing ~70% sequence identity [1,6,73,74,75,76,77]. NCX1 is a ubiquitous isoform; although its numerous splice variants (at least 17) are expressed in a tissue-specific manner (e.g., only one isoform/splice variant, NCX1.1, is expressed in cardiomyocytes), these variants modulate cardiac excitation-contraction coupling, brain potentiation, kidney, and intestinal Ca^2+^ absorption, bone formation, endothelial tonus, and pancreatic hormonal secretion, among many other functions [6,26,73] (Figure 2B). NCX2 does not undergo splicing and is preferentially expressed in the brain, spinal cord, and gastrointestinal and kidney tissues [74,75,76,77]. At least five splice variants of NCX3 are expressed in the neuronal and smooth muscle tissues while contributing to stress conditions (neuronal excitotoxicity, brain stroke, and neuronal injuries), slow-twitch muscle contraction, and long-term potentiation in the hippocampus [10,23,78]. Significant levels of NCX3 are also found in the bone and glandular epithelial cells, suggesting that NCX3 plays a vital role in bone formation and hormone secretion [79,80]. The functional roles of NCXs were discussed in recent publications [6,23,78,81] and will not be discussed here.

## 3. The Ca^2+^/CA Antiporters Translocate Ca^2+^ in Exchange for Different Counter-Ions

Ion-transporting proteins (channels, transporters, and pumps) selectively recognize H^+^, Na^+^, K^+^, Ca^2+^, and Mg^2+^ ions while each ion-transporting group undergoes characteristic conformational changes to mediate ion transport [43,44] Understanding how ion recognition events can be coupled with transport machinery remains challenging. The superfamily of the Ca^2+^/CA antiporters represents a fascinating group of structurally related proteins, which can transport Na^+^, K^+^, H^+^, Li^+^, and perhaps some other ions in exchange with Ca^2+^. The major challenge is to identify, segregate, and characterize structure-dynamic determinants that can predefine ion selectivity at multiple sites as well as resolve how the ion interactions with respective sites can induce alternating access of the ion-binding pocket [31,32,33]. In this respect, the fundamental paradigm of transporter function describes the alternative access (exposure) of the substrate (ion) binding sites to either one side of the membrane or the other during the transport cycle [26,31,32,33]. According to this fundamental paradigm, the ligand (ion) transporter protein must undergo the inward-facing (IF) and outward-facing (OF) conformation states during the transport cycle [26,31,32]. The structure-dynamic transitions associated with the swapping of the OF and IF states might involve numerous intermediates. The identification and functional assignment of involved intermediates remain challenging even for the most studied proteins in the field. In any case, it is fully appreciated that the underlying mechanisms may predefine biologically important features (e.g., ion selectivity, transport rates, ion-binding affinities at opposite sides of the membrane, electrogenic responses to varying membrane potential among many others) [3,26,31,32]. In general, the swapping of the IF and OF states may occur either in the presence or absence of ligand (ion), whereas the structural nuances of the alternating access mechanism differ considerably among different categories of secondary transporters [31,32]. For example, in the cotransporter system, the OF/IF swapping can occur either in the presence or absence of the ligand (ion), whereas in the antiporter system (such are the Ca^2+^/CA proteins) the ligand (ion) interaction with respective sites is mandatory to promote the OF/IF swapping [31,32,33]. Thus, the emerging challenge is to elucidate structure-dynamic determinants associated with the ion-induced swapping of the OF/IF states in distinct Ca^2+^/CA families, each having a characteristic ion selectivity.

### 3.1. The NCX_Mj Structure as a Prototype Model for Studying Ion-Transport Mechanisms

**Figure 3:**
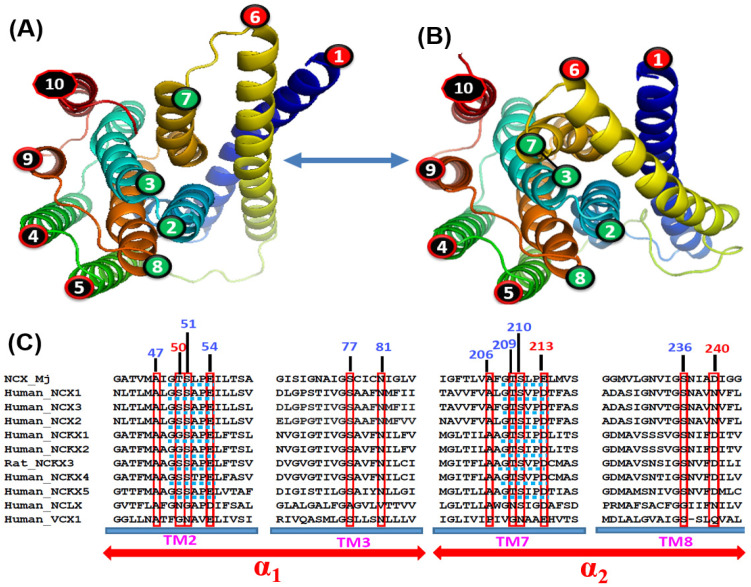
Structural organization and sequence alignment of ion-coordinating residues in NCXs and similar proteins. The sliding mechanism is a distinct variant of the alternating access mechanism while describing specific conformational changes associated with the OF/IF swapping. The open (X-ray structure) (**A**) and closed (computed) (**B**) structures of NCX_Mj at the extracellular side. Upon ion binding, the two-helix sliding bundle, TM1/TM6 (presented in red), might relocates in front of other helixes to swap the OF and IF states. (**C**) Sequence alignment of NCX, NCKX, NCLX, and CAX antiporters (belonging to the superfamily of Ca^2+^/CA antiporters). Twelve ion-coordinating residues (in red boxes), attached to four helixes (TM2, TM3, TM7, and TM8) form an ion-passageway entity. The ion-coordinating residues are highly conserved in Ca^2+^/CA proteins—e.g., the NCX_Mj and mammalian NCXs have three (out of twelve) different ion-coordinating residues (T50S, E213D and D240N) although their transport rates differ by several orders of magnitude.

The breakthrough discovery of the crystal structure of archaeal *Methanococcus jannaschii* NCX (NCX_Mj) provided new opportunities for structure-based biophysical studies aiming to resolve the mechanisms underlying ion transport [27,28]. The NCX_Mj structure may represent a unifying model for investigating the ion-transport mechanisms [3,4,24,25] since the ion-coordinating residues (at transport sites) are highly conserved among the prokaryotic and eukaryotic NCX orthologs (Figure 3C). However, in contrast with eukaryotic NCXs, NCX_Mj lacks regulatory domains [37,38,39,40,41,42]. The follow-up complementary studies by using MD simulations, e.g., HDX-MS, ATR-FTIR, and 2D IR techniques, in conjunction with an extended analysis of the mutational effects on the ion-transport rates, provide a wealth of information on the structure-dynamic features underlying the ion transport events in NCX_Mj [60,68,69,70,71,72,82,83].

### 3.2. Structural Bases of Ion Transport Stoichiometry, Selectivity, and Alternating Access

High-resolution crystal structures of NCX_Mj capture the outward-facing (OF) conformation, where four ion-binding sites (S_ext_, S_mid_, S_int_, and S_Ca_) form a diamond-shaped configuration [27,28] (Figure 1E,F). According to these structures, the S_int_ and S_ext_ sites have high selectivity for Na^+^, whereas the S_Ca_ and S_mid_ sites show no preferential selectivity for either Na^+^ or Ca^2+^. Initially, it was proposed that the 3Na^+^ ions occupy the S_ext_, S_mid_, and S_int_ sites, whereas 1Ca^2+^ occupies the S_Ca_ site, thereby suggesting that 1Ca^2+^ and 3Na^+^ occupy entirely different sites in a mutually exclusive way [27]. The follow-up studies with MD simulations and analyses of the mutational effects on the ion fluxes assigned an alternative occupation of S_int_, S_ext_, and S_Ca_ by 3Na^+^ or the occupation of S_Ca_ by 1Ca^2+^ [82]. In agreement with this model, the follow-up crystallographic studies underscored the unique features of the S_Ca_ site, which can be alternatively occupied by either Na^+^ or Ca^2+^ [28]. This revised model was further supported by HDX-MS, ATR-FTIR, and 2D IR analyses [68,69,70,71,72]. Despite this progress, the functional status of the S_mid_ site remains unclear. The MD simulations and X-ray studies suggest that S_mid_ may become occupied by a water molecule through protonated D240 in the ground state [28,82]. Although the S_mid_ site cannot bind either Na^+^ or Ca^2+^ (at least in the OF ground state), this site may contribute to the stabilization of the ion-bound transition state when the OF/IF swapping occurs [60,68,72]. Alternatively, the Na^+^ or Ca^2+^ ion may occupy the S_mid_ site in the IF ground state, although the IF crystal structure of NCX_Mj is required to validate this proposal.

Structure-based functional assignment of ion binding sites, using the prototype models of NCX, CAX, NCKX, and NCLX proteins [27,28,33,34,35,36,45,46,47,72,82], strongly support the notion that ion selectivity distinctions at multiple sites predefine the ion-exchange stoichiometry for a given family of antiporters. Despite these differences in ion selectivity and ion exchange stoichiometry and transport rates, the Ca^2+^/CA proteins might share a common mechanism of ion-induced alternating access, the structure-dynamic details of which remain to be validated (see below).

### 3.3. The Ca^2+^/CA Proteins Might Share a Common Mechanism of Alternating Access

Like many other secondary transporters, the Ca^2+^/CA proteins comply with the fundamental mechanism of alternating access, according to which the ligand (ion) binding pocket of a given protein undergoes alternative exposures (accesses) at opposite sides of the membrane while adopting the inward-facing (IF) and outward-facing (OF) states in succession [60,68,72,82]. High-resolution crystal structures of NCX_Mj in the OF state [27,28] and CAX proteins in the IF state [34,35,36], in conjunction with biophysical studies [60,68,69,70,71,72,82,83], have elucidated mechanistic aspects of ion transport. Despite this progress, it remains unclear how the occupation of multiple sites (possessing a different ion selectivity) can induce conformational changes that manage the OF/IF swapping.

#### 3.3.1. Ion Binding Sites of CAX

The high-resolution crystal structures of the H^+^/Ca^2+^ exchanger proteins (the CAX family), depict the open, semi-open, and occluded states [34,35,36]. It was suggested that the CAX proteins contain the mutually exclusive sites for 1Ca^2+^ (S_Ca_) and 2H^+^ (S_ext_ and S_int_) binding, consistent with the ion-exchange stoichiometry of 2H^+^:1Ca^2+^. Unfortunately, no efforts have been made to measure the ion-exchange stoichiometry of CAX proteins. This missing information is somewhat irritating since according to the current models of the CAX proteins [34,35,36], the ion selectivity features of the S_Ca_ site in CAX are principally different from the ion selectivity features of the S_Ca_ site in NCX_Mj [28,82], NCKX [45,46,47] or NCLX [84]. More specifically, according to the existing CAX models, the S_Ca_ site of CAX binds Ca^2+^ (but not the counter ion, H^+^), whereas the matching models of NCX, NCKX and NCLX revealed that the S_Ca_ site in these proteins can bind either the Ca^2+^ or Na^+^ ion [28,47,84]. It is worthwhile to note that according to the unifying mechanism of ion-induced coupling of ion transport in the Ca^2+^/CA proteins, the S_Ca_ site of CAX has to bind either Ca^2+^ or H^+^. More dedicated experimentation is required to examine this proposal.

#### 3.3.2. Ion Binding Sites of NCKX

The NCKX proteins mediate the K^+^-dependent Na^+^/Ca^2+^ exchange by transporting 1Ca^2+^ + 1K^+^ in exchange for 4Na^+^ ions [45,46,47]. The differences in ion selectivity and the ion-exchange stoichiometry between the NCXs (3Na^+^:1Ca^2+^) and NCKXs (4Na^+^:1Ca^2+^,1K^+^) are quite amazing since ten (out of twelve) ion-coordinating residues are identical in NCX_Mj and NCKX proteins (Figure 3C). Based on the crystal structure of NCX_Mj, the MD simulations and extended analyses of mutational effects on the ion fluxes led to the conclusion that four putative sites of NCKX can be alternatively occupied by 4Na^+^ (at S_int_, S_mid_, S_ext_ and S_Ca_) or 1Ca^2+^ (at S_Ca_) + 1K^+^ (at S_mid_) [45,46,47]. Structure-based computational analyses of ion selectivity at four sites of NCX_Mj revealed that a simultaneous occupation of all four sites of NCX_Mj by 4Na^+^ is thermodynamically forbidden [82]. These findings underscore a fundamental disparity between NCX and NCKX proteins in ion selectivity and ion binding/transport stoichiometry. However, it remains unclear how two ion-coordinating residues (T50S and E213D) can diversify the ion selectivity profiles of S_mid_ in NCX and NCKX. Collectively, the ion-selectivity features of the S_mid_ site do account for functional differences between NCX and NCKX proteins. Namely, the S_mid_ site of NCX_Mj cannot bind Na^+^, Ca^+^, or K^+^ at any stage of ion binding/translocation, whereas the S_mid_ site of NCKX can alternatively bind either Na^+^ or K^+^. Even though the functional significance of T50S and E213D (and other ion-coordinating residues) were experimentally resolved for a native NCKX [45,46,47], some additional physical factors (e.g., a space hindrance, water accessibility, and hydrophobicity within the S_mid_ site vicinity) may shape the ion selectivity of S_mid_ in NCKX. Structural discovery of full-size NCKX may resolve these issues.

#### 3.3.3. Ion Binding Sites of NCLX

Even though, both the plasma membrane (NCX) and the mitochondrial (NCLX) proteins mediate the Na^+^/Ca^2+^ exchange under physiologically related conditions, NCLX lacks the regulatory CBD1 and CBD2 domains. Moreover, NCX and NCLX exhibit different ion selectivity profiles [85,86]. Namely, NCX possesses high selectivity for Na^+^ or Ca^2+^, whereas NCLX can transport either Li^+^ or Na^+^ in exchange for Ca^2+^ [85]. In contrast with NCX_Mj, the stoichiometry of NCLX-mediated ion exchange as well as the structure-function assignment of the ion selectivity features at respective sites remains elusive due to the lack of structural information [48,87,88]. To elucidate the underlying mechanisms of ion recognition/transport in mitochondrial NCLX, the Li^+^-transporting NCLX_Mj chimera was generated by replacing the ion-coordinating residues in the archaeal NCX (NCX_Mj) to imitate the structural organization of ion-coordinating residues assigned to human mitochondrial NCLX [89,90]. In contrast with the parent protein (NCX_Mj), the newly generated construct (NCLX_Mj) mediates both the Na^+^/Ca^2+^ and the Li^+^/Ca^2+^ exchange modes, as the native NCLX does [85,86,87,88,89,90]. Thus, NCLX_Mj can serve as a reasonably good model for studying the ion transport mechanisms of native NCLX.

The MD simulations revealed that NCLX_Mj contains two Li^+^ (or Na^+^) binding sites with four ion-coordinating residues, where these two brand-new sites are derived from the three Na^+^ binding sites of NCX_Mj, thereby generating the electroneutral Na^+^/Ca^2+^ or Li^+^/Ca^2+^ exchange reactions [84]. This model was further supported by 2D IR spectroscopy and mutational analyses of ion fluxes, thereby suggesting that a native mitochondrial NCLX may mediate the electroneutral ion-exchange modes (2Na^+^:1Ca^2+^ or 2Li^+^:1Ca^2+^) [84]. Thus, in contrast with NCX and NCKX (which contain either the three and four sites for monovalent ion binding, respectively) two sites of NCLX bind either 2Na^+^ or 2Li^+^. According to the NCLX_Mj model, only four ion-coordinating residues (N50, D54, N209, and D213) contribute to multi-dentate ion ligation, where the side-chains of D54 and D213 and the backbone carbonyl oxygens of N50 and N209 coordinate either 2 Li^+^ or 2Na^+^ [84]. Thus, the ion-coordination module of NCLX_Mj can alternatively bind to either 1Ca^2+^ or 2Na^+^/2Li^+^ ions at different steps of the transport cycle, while generating the electroneutral ion-exchange cycle (in contrast with NCX proteins). Although this new structure-functional model of NCLX_Mj requires further confirmation for native mitochondrial NCLX, the currently available experimental findings are difficult to reconcile with 3Na^+^ or 3Li^+^ binding sites. Since the S_Ca_ sites of NCX, NCKX and NCLX share common ion selectivity and ion-ligation modes, this site may provide a structural basis for a common mechanism of ion-induced alternating access in Ca^2+^/CA proteins.

### 3.4. Structure-Dynamic Basis of Functional Asymmetry in NCX_Mj and Similar Proteins

The available crystal structures of NCX_Mj were obtained for ion-bound species showing the open, semi-open, or occluded conformation in the OF state [27,28]. In contrast with NCX_Mj, the ion-bound species of the CAX proteins exhibit distinct conformational states, while favorably accommodating the IF state [34,35,36]. Since the crystallization of apo NCX_Mj remains challenging (at least at physiologically relevant pHs), the question arises of whether the structure-functional asymmetry is an intrinsic feature of the apo NCX_Mj protein or whether ion interactions with specific sites are required to create lopsided access of ions to the extracellular and cytosolic vestibules under steady-state conditions. To address this question, the site-directed covalent labeling of the extracellular and cytosolic vestibules was done in conjunction with HDX-MS analysis [68,69]. These experiments have identified characteristic differences in the backbone dynamics at the extracellular and cytosolic vestibules while highlighting conformational differences between the OF and IF states. It was concluded that the structure-dynamic differences in the OF and IF states are predefined by apo-protein structure, even though the Na^+^ or Ca^2+^ binding results in incremental conformational changes at specific locations [68,69,70,71,72]. The HDX-MS analysis of apo and ion-bound species, combined with mutational analysis of ion fluxes, have identified specific structural elements that contribute to a preferential stabilization of the OF state vs. the IF state [60,68,69,70,71,72]. For example, the 196-DSK-198 segment can stabilize the interface between TM6 and TM7 through the hydrogen bonding network, thereby affecting the rate-equilibrium relationships of bidirectional ion movements [3,8,60]. Since the relevant structural-functional relationships were discussed in recent publications [24,25,26,60,69,72], these issues will not be discussed here.

### 3.5. Structure-Dynamic Specificities Associated with Ion Occlusion in NCX_Mj

**Figure 4:**
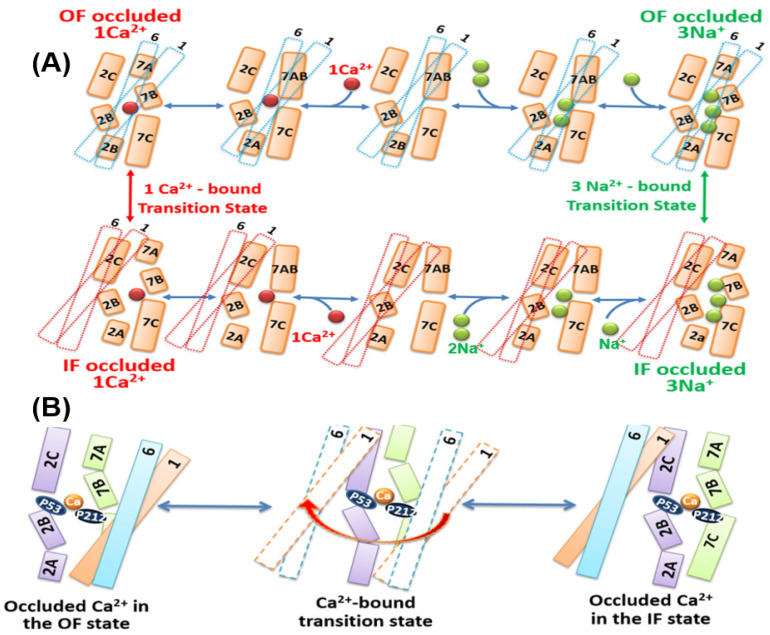
The Ca^2+^- and Na^+^-bound intermediates during the transport cycle of Na^+^/Ca^2+^ exchange. (**A**) The Na^+^/Ca^2+^ exchange cycle involves separate steps of 3Na^+^ or 1Ca^2+^ translocation across the membrane where the transition state (either for Na^+^ or Ca^2+^ transport step) is between the occluded states at the extracellular and cytosolic sides. According to this model, the TM1/TM6 undergoes a sliding movement toward performing the OF/IF swapping. Ion interactions with respective sites generate the occluded states either at the extracellular or cytosolic side (green and red spheres represent the Na^+^ and Ca^2+^ ions, respectively). Dashed lines refer to the two-helix sliding bundle, TM1/TM6). (**B**) The emerging working hypothesis is that the occupation of the S_Ca_ site plays a critical role in closing the hydrophilic gap between the TM2C (P53) and TM7B (P212) segments, thereby forming a transient hydrophobic patch. The transient hydrophobic environment between TM2 and TM7 can facilitate the sliding movement of the TM1/TM6 bundle on the protein surface, thereby accomplishing the OF/IF swapping.

High-resolution X-ray data, combined with well-suited procedures of MD simulations, demonstrated that upon the occupation of high-affinity binding sites (S_int_ and S_Ca_) by 2Na^+^ ions (which takes place at low concentrations of Na^+^), the NCX_Mj protein in the OF state is captured while adopting a semi-open conformation [28]. At high Na^+^ concentrations, the binding of the third Na^+^ to the low-affinity S_ext_ site results in a subtle backbone bending at the interface of the TM7A and TM7B segments, which occludes 3Na^+^ ions in the OF state. The follow-up HDX-MS experiments, combined with mutational analyses of ion fluxes revealed that Na^+^ binding to S_ext_ results in the bending of the TM7 segment into two short helices (TM7A and TM7B) [68,69]. According to this model, the coordination of the third Na^+^ ion at S_ext_ through the backbone carbonyl of A206 and the bulk aromatic ring of F202 prevents the dissociation of all 3Na^+^ ions from the extracellular vestibule (Figure 4A).

Because of the lack of the NCX_Mj crystal structure in the IF state, the mechanisms underlying ion occlusion at the cytosolic side remain unresolved. The HDX-MS studies, combined with computational approaches and mutational analyses of ion fluxes, have detected some specific conformational changes that could be associated with the ion occlusion events in the IF state at the cytosolic side [68,69]. More dedicated analyses of nanodisc-reconstituted preparations of NCX_Mj by using the advanced approaches of HDX-MS [91,92,93] and ^19^F-NMR [94,95,96] may provide valuable information on the ion-induced conformational changes in NCX_Mj and similar proteins.

### 3.6. Structural Elements Associated with the Unifying Mechanism of Alternating Access

The crystal structures of NCX_Mj in the OF conformation reveal a tightly packed core of eight helices (TM2-TM5 and TM7-TM10), where two long and loosely packed helices (TM1 and TM6) form a tilted bundle (TM1/TM6) in front of a rigid eight-helix core [27,28] (Figure 1C,D and Figure 3A,B). This structural information put forward the ‘sliding mechanism’ of alternating access, according to which the ion-induced sliding of the TM1/TM6 bundle toward the rigid eight-helix core represents a major conformational change that occurs during OF/IF swapping. The follow-up crystal structures of CAX proteins were obtained in the IF state; they exhibit striking structural similarities to the NCX_Mj structure in the OF state [34,35,36]. Based on these structural data, it was proposed that the Ca^2+^/CA proteins possess a common mechanism of alternating access, although it remains unclear how the occupation of different binding sites (with diverse ion selectivity) can induce TM1/TM6 sliding on the protein surface. In the case of CAX_Af, it was suggested that the binding of Ca^2+^ and H^+^ to separate sites results in narrowing (compression) of the central pore, where the resulting closure of the gap between TM2 and TM7 forms a hydrophobic patch, which can allow a favorable sliding of the TM1/TM6 bundle toward the OF/IF swapping [35]. Consistent with this proposal, the HDX-MS and mutational studies revealed that Na^+^ or Ca^2+^ interactions with NCX_Mj rigidify the local backbone dynamics at TM2C (P53) and TM7C (P212), nearby the external interior of the S_Ca_ site [68,69,72].

Collectively, the currently available data support the notion that occupation of the S_Ca_ site by either Na^+^ or Ca^2+^ attracts the flexible segments (TM7B, TM7C, TM2C, and TM8A) to the rigid TM2B segment. These subtle conformational changes (associated with the occupation of the S_Ca_ site by either Na^+^ or Ca^2+^) may result in a transient gap closure between TM2 and TM7 with a subsequent formation of a hydrophobic patch. The transient formation of this hydrophobic parch can facilitate the sliding of the TM1/TM6 bundle on the protein surface, thereby allowing the OF/IF swapping (Figure 4B). More dedicated research is required to resolve the structure-dynamic details underlying the dynamic transitions between the OF and IF states.

### 3.7. Structure-Dynamic Causes of Kinetic Variances Remain to Be Resolved

The vast differences in transport rates, observed between NCX_Mj and cardiac NCX1.1 [55,56,57,58,59,60] are very remarkable since they share a common stoichiometry for ion exchange [51,52,53]. This is may have a fundamental significance since phylogenetically distinct NCX orthologs contain highly conserved repeats (α_1_ and α_2_) with very similar ion-coordinating residues (Figure 3A,B). Moreover, the differences in the ion transport rates between NCX_Mj and NCX1.1 are remarkable since only three (out of twelve) ion-coordinating residues (T50S, E213D and D240N) differ between the archaeal NCX_Mj and mammalian NCX1-3 variants (Figure 3C). Thus, one may posit that the differing ion-coordinating residues account (at least partially) for the kinetic differences. To test this hypothesis, the structure-based replacements of relevant ion-coordinating residues performed in NCX_Mj to evaluate how the residue replacements at matching positions can imitate the anticipated kinetic values possessed by native eukaryotic NCXs [67]. In this experimental setup, the single (T50S, E213D, and D240N), double (T50S/E213D and T50/D240N), and triple (T50S/E213D/D240N) mutations of NCX_Mj were tested for their effects on the *k*_cat_ and K_m_ values of the Na^+^/Ca^2+^ or Ca^2+^/Ca^2+^ exchange reactions. Strikingly, the K_m_ and *k*_cat_ values of NCX_Mj mutants are comparable to the kinetic parameters of the parent NCX_Mj, whereas the transport rates of the cardiac NCX1.1 are by several orders of magnitude higher than in the parent NCX_Mj or mutants [60,67]. These data strongly support the notion that the structure-based replacements of relevant ion-coordinating residues (T50S, E213D, and D240N) in NCX_Mj cannot recapitulate (even partially) the anticipated transport rates possessed by mammalian NCXs, although a given mutant contains all twelve ion-coordinating residues of mammalian NCXs. Since the archaeal proteins are known for their thermophilic nature, the intrinsic rigidity of NCX_Mj may restrict ion transport rates, even though all catalytic residues are there.

The kinetic analysis of NCX1.1 revealed that the transport of Na^+^-bound species (during the forward mode of the unidirectional ion transport) is a rate-limiting step throughout the Na^+^/Ca^2+^ exchange cycle [56,58,97,98,99]. Specific structural elements may affect the kinetics of the rate-liming step, thereby influencing the rate-equilibrium relationships of the ion exchange cycle. For example, the elongation of the cytosolic 5L6 loop of NCX_Mj (by 8 or 14 residues) accelerates the ion-exchange rates 7–10-fold and shifts the steady-state OF/IF equilibrium from 0.2 to 15–35, thereby favorably stabilizing the IF state [60,69,70,84]. Most probably, the elongation of the 5L6 loop generates more flexible (less restricted) conformational states for relocating the TM1/TM6 bundle, thereby accelerating the rate-limiting step of 3Na^+^ occlusion, which precedes the OF/IF swapping. More dedicated research is required to identify and resolve structural elements that control the kinetic performance of NCX orthologs, isoforms, and splice variants.

Besides the structure-dynamic features of NCXs, some external factors may affect the transport rates as well. For example, negatively charged phospholipids and cholesterol activate (up to 10–20-fold) the NCX1.1-mediated transport rates [100,101], whereas NCX_Mj shows no response to varying compositions of lipids [67]. Thus, the lipid-dependent effects may account (at least partially) for kinetic disparities between NCX_Mj and NCX1.1. In light of the recent discoveries revealing that specific lipid–protein interactions predefine functional performances of Na^+^/K^+^ ATPase and other membrane proteins [102,103]. This underscores an emerging need in investigating lipid–protein interactions in distinct mammalian variants of NCX (see Section 6, below).

### 3.8. Charge-Carrying Features of Ion-Bound Species Are Alike among NCXs

Structural studies of prokaryotic NCX_Mj and the sequence alignments of eukaryotic NCXs have established that the ion-binding pocket of NCX_Mj (E54, E213, and D240) and mammalian (E54 and D213) NCXs contain either three or two carboxylates, respectively (Figure 2). In general, these differences in the number of carboxylate residues may contribute to the huge kinetic differences between the prokaryotic and eukaryotic NCXs. Thus, one may propose that at deprotonation of all ion-coordinating carboxylates, the 3Na^+^-bound species must be electroneutral (Z = 0) in NCX_Mj, whereas the 1Ca^2+^-bound species must bear a negative charge (Z = −1). In sharp contrast with this proposal, MD simulations and extended tests of the mutational effects on the transport rates strongly support a model according to which E54 and E213 are deprotonated, whereas D240 is protonated (at least at physiological pH values) [70,72,82]. According to this model, the 3Na^+^-bound species of NCX_Mj carry a positive charge (Z = +1), whereas the 1Ca^+^-bound species are electroneutral (Z = 0). Even though the mammalian NCXs contain only two carboxylates for ion coordination/transport, the kinetic analysis of ion fluxes revealed that the translocation of positively charged 3Na^+^-bound species (e.g., by cardiac NCX1.1) involves a voltage-sensitive (and rate-limiting) step during the transport cycle, whereas the translocation of Ca^2+^-bound species is voltage insensitive [56,58,97,98,99,104]. Thus, the currently available data are consistent with the notion that two deprotonated carboxylate residues are involved in 3Na^+^ or 1Ca^2+^ ligation, either in NCX_Mj or NCX variants. According to this proposal, 3Na^+^-bound species might carry a positive charge (Z = +1), whereas the 1Ca^+^-bound species might be electroneutral (Z = 0) either in NCX_Mj or eukaryotic NCXs. Thus, the differences in the number of carboxyl residues within the ion-binding pocket cannot account for kinetic differences between NCX_Mj and NCX1.1.

## 4. Regulatory Divergence of NCXs Is Required to Match Cell-Specific Ca^2+^ Signaling

In general, two major trends of allosteric regulation operate in eukaryotic NCXs: (1) ion-dependent regulation by cytosolic Na^+^, Ca^2+^, and H^+^ ions and (2) ligand-dependent (metabolic) regulation by ATP, PIP_2_, phosphoarginine, phosphocreatine, and other ligands [29,63,105,106,107,108,109,110,111,112]. Genetically encoded structural variances in isoform/splice variants of mammalian NCX (expressed in a tissue-specific manner) exhibit characteristic differences in ion- and/or ligand-dependent regulation (Figure 2A,B) [81,105,106,107,108,109,110,111,112,113]. In potential, the structure-based development of cell-specific NCX blockers and activators may provide game-changing pharmacological tools for treating and preventing pathophysiological conditions [10,11,12,13,14,15,16,17,18,19,20,21]. Structure-based biophysical studies of isolated regulatory domains [112,114,115,116,117,118,119,120,121,122,123,124,125] and patch-clamp analyses of full-size NCX variants [39,40,126], disclosed the basic and editing mechanisms underlying the regulatory specificities possessed by NCX variants [3,6,8,26]. Despite this progress, it remains unclear how the allosteric messages are decoded and transferred from the regulatory to the transport domains (over a distance of ~80 Å) and how the allosteric signals become integrated in a given NCX variant.

### 4.1. NCX Coupling with Other Ion Transport Systems Requires a Further Resolution

The Na^+^ entry through a given ion channel and transporter can activate the reverse (Ca^2+^ entry) mode of NCX, which plays a critical role in controlling the directionality of Na^+^ and Ca^2+^ net fluxes to support cell-specific functional swings [2,6,8,12,14,29]. This functional coupling between NCX and other ion-transporting systems may occur under physiological or pathophysiological conditions [2,9,12,14,127,128,129,130,131,132,133,134]. The Na^+^-entry promoted reverse mode of NCX chiefly contributes to the Ca^2+^-influx in diverse cell types to couple the Ca^2+^ and Na^+^ transients in neuron-glial cells, excitation-contraction-coupling event in cardiac and skeletal myocytes, the strength of vascular constriction, and dynamic features of synaptic transmission among many others [9,12,14,127,128,129,130,131,132,133,134]. There has been increasing interest in NCX operation regulated by coupling or interaction with other cation channels; such are TRP, TRPC3, and TRPC6 [9,127,128,129,130,131,132,133,134]. For example, recent studies revealed that NCX1 is a functionally important partner of STIM1 in mediating SOCE since the activation of the NCX1 reverse mode can be promoted by a local increase of [Na^+^] through STIM1 and TRPC6 coupling [128]. The NCX-mediated Ca^2+^-influx usually occurs when a rapid Na^+^-influx (through a given channel or transporter) elevates [Na^+^]_i_ within a restricted space [128,134,135,136]. For example, in smooth muscle cells, the Ca^2+^-entry through NCX1 is coupled with Na^+^ transport via store-operated channels, TRPC6 and Orai1, where TRPC6, Orai1, and NCX1 are co-localized with the α-2 Na^+^/K^+^ ATPase to form a structural cluster within the cell (plasma) membrane nearby the SR junction [128,129,134,135,136,137,138]. The question is how the transported ions can reach a target sink without “ion escape” to the cytosol bulk phase. To resolve this problem, it is essential to obtain detailed information on physical factors (viscosity, site density, nanodomain space, and configuration among others) that can limit ion diffusion within a restricted space. Moreover, the dynamic coupling between the NCX and other proteins might occur within a restricted space and time window (e.g., during the action potential), where the regulatory (allosteric) effects of Ca^2+^ and Na^+^ on NCX can characteristically vary pending on a given NCX variant.

The underlying mechanisms of Ca^2+^ signaling/homeostasis in mitochondria have been studied extensively during the past few years, and several molecular players involved in mitochondrial Ca^2+^ uptake and extrusion have been identified. More than a decade ago, it was proposed that the mitochondrial Letm1 protein (originally discovered as mitochondrial K^+^/H^+^ exchanger) extrudes Ca^2+^ from mitochondria through the H^+^/Ca^2+^ exchange mode although the functional significance of this mode in mitochondrial Ca^2+^ extrusion has been questioned [48,88,139,140,141]. The molecular identification of NCLX [86] and numerous follow-up investigations revealed that Na^+^-dependent Ca^2+^ release through NCLX represents a major pathway for mitochondrial Ca^2+^ extrusion (accounting for ~75%), whereas the Na^+^-independent Ca^2+^ release pathways from mitochondria may account for the rest 20–25% [48,86,88]. Recently, it was identified that TMBIM5 (but not Letm1) is the long-sought mitochondrial transporter that can mediate the H^+^/Ca^2+^ exchange while controlling a proton gradient [142,143]. The emerging working hypothesis is that specific protein–protein interactions (involving TMBIM5 and other proteins) may control mitochondrial Ca^2+^, K^+^, and H^+^ signaling homeostasis and mitochondrial proteomes under normal and pathophysiological conditions [88,142,143,144]. Even though new findings underscore molecular mechanisms that can integrate mitochondrial ion signaling/homeostasis, metabolism, bioenergetics, and proteomes, NCLX is a predominant and universal mechanism for mitochondrial Ca^2+^ extrusion in various cell types [48,88,144,145,146,147,148].

Mitochondrial localization of NCX1, NCX2, and NCX3 isoforms reported, suggesting that NCX associates with the outer membrane of mitochondria [149,150,151,152,153]. According to this proposal, the Ca^2+^ extrusion from mitochondria involves two sequential steps where NCLX primarily extrudes Ca^2+^ from the mitochondrial matrix to the intermembrane space, whereas NCX subsequently removes Ca^2+^ from the intermembrane space to the cytosol [150,151,152,153]. Two critical points should be carefully considered in respect with this proposal. Firstly, the outer membrane of mitochondria is highly permeable to cations (including Ca^2+^ and Na^+^), so the functional relevance of the NCX location in the outer membrane of mitochondria is not trivial to rationalize (at least at this stage of our knowledge). Secondly, NCX and NCLX have distinct signal peptide sequences (at the N-terminus), which predefine compartmental incorporation of NCX or NCLX into the cell (plasma) membrane or into the inter membrane of mitochondria, respectively—thus, a proposed incorporation of NCX into the outer membrane of mitochondria is difficult to substantiate. Despite these critical points, it is essential to note that the palmitoylation-dependent incorporation of NCX into the ER membrane [154,155,156,157,158,159,160,161,162] could be relevant for developing feasible approaches. For example, the EM-associated NCX may interact with a MEM-protein network (at the contact interphase of EM/mitochondria), since this sophisticated molecular complex can control the ER Ca^2+^ stories although the underlying mechanisms are incompletely understood [163,164,165,166]. One may posit that the MEM-associated NCX may replenish the ER Ca^2+^ stories under stress conditions in response to specific signals that are coming from mitochondria. This putative mechanism may underscore the role of NCX in the delay of ER stress and cell death during the early phase of neurodegenerative diseases [150,151,152,153,163,164,165,166]. In light of the present considerations, it is required to resolve the underlying mechanisms of NCX involvement in coupling the ER and mitochondria interactions as related to the control of ER Ca^2+^ levels in health and disease.

### 4.2. Eukaryotic NCXs Exhibit Different Modes of Ion-Dependent Regulation

The mammalian and invertebrate NCX orthologs/isoform/splice variants are chiefly regulated by cytosolic Ca^2+^, Na^+^, and H^+^ ions, although each given variant exhibits structure-based characteristic profiles of ion-dependent regulation [29,77,105,106,107]. In general, cytosolic Ca^2+^ activates all isoform/splice variants of NCX through allosteric interactions [52,77,99,120], whereas the H^+^ and Na^+^ ions have an inhibitory effect [29,77,105,120]. For example, the tissue-specific splice variants of NCX1 possess Na^+^-dependent inactivation, although in the cardiac (NCX1.1) and brain (NCX1.4) variants the Na^+^-dependent inactivation can be alleviated by cytosolic Ca^2+^ and/or ATP/PIP_2_, whereas the kidney (NCX1.3) variant lacks the Ca^2+^-dependent alleviation of Na^+^-induced inactivation [77,81,167,168,169]. In general, Ca^2+^ binding to CBD1 activates mammalian NCXs, whereas Ca^2+^ binding to CBD2 alleviates the Na^+^-induced inactivation [39,40,120]. Squid axon and mammalian NCXs (but not NCX_Mj) exhibit high sensitivity to cytosolic pH changes, where even slight acidification of the cytosolic pH from 7.2 to 6.9 results in ~90% inhibition of NCX-mediated ion currents [29,63,64]. This phenomenon, known as a proton block, is of general interest since proton-dependent inactivation of NCX may operate under acidosis/ischemia conditions to prevent NCX-mediated arrhythmogenicity due to Ca^2+^ overload [63].

#### 4.2.1. NCX and CALX Exhibit Positive, Negative, or No Response to Regulatory Ca^2+^

A physiologically relevant transient uplift in cytosolic [Ca^2+^]_i_ can raise the mammalian NCX currents ~25-fold, whereas the removal of cytosolic Ca^2+^ results in a slow inactivation termed I_2_-inactivation or Ca^2+^-dependent inactivation [52,57,120]. The Ca^2+^-dependent increase in peak current occurs at lower [Ca^2+^] levels (~0.2 μM) [116,117,118,119,120,121,122,123,124,170] while showing an exceptionally high degree of cooperation with a Hill coefficient of n = 4–8 [57,120]. Thus, the Ca^2+^-dependent activation of the cardiac NCX1.1 (for example) during the action potential (when the cytosolic Ca^2+^ concentrations rapidly rise 10–20-fold) represents a major regulatory mode for NCX activation under physiologically related conditions. Notably, the Ca^2+^-dependent alleviation of Na^+^-induced inactivation requires relatively higher Ca^2+^ concentrations (5–100 μM) either for NCX1 or NCX3 [105,106,107,108,109,110,111,112,169]. The patch-clamp analyses of full-size NCX mutants [39,40] and the Ca^2+^-binding assays in isolated CBD12 preparations [116,117,118,119,120] demonstrated that the high-affinity Ca^2+^ sites of CBD1 are responsible for the mammalian NCX activation, whereas the Ca^2+^-dependent alleviation of Na^+^-induced inactivation is instigated by low-affinity Ca^2+^ binding to CBD2. Note that the NCX1 splice variants containing exon B and the NCX3 splice variants containing exon A cannot perform the Ca^2+^-dependent alleviation of Na^+^-induced inactivation, since there is no Ca^2+^ site at CBD2 in these splice variants [3,8,26,38,116,117,118,119].

In sharp contrast to mammalian NCXs, a *Drosophila* NCX (CALX1) exhibits opposite regulatory responses to cytosolic Ca^2+^. In the CALX1.1 splice variant, the Ca^2+^ binding to CBD1 inactivates the transport rates, whereas the Na^+^ binding to some unknown site activates the transport rates [171,172,173]. Although the Ca^2+^ binding to CBD1 of CALX1.1 inhibits ion transport, the Ca^2+^ has no regulatory effect on the ion transport in CALX1.2 [171,172,173]. An open question is: how could it be possible that Ca^2+^ binding to CBD1 activates (mammalian NCXs), inhibits (CALX1.1), or does not affect (CALX1.2) the ion-exchange rates? This is a very peculiar question since the structural organization of Ca^2+^ ligation at CBD1 is highly conserved among NCX and CALX orthologs, isoforms, and splice variants [39,40,41,42,172,173]. The emerging working hypothesis is that some minute (albeit very characteristic) disparities in the structure-dynamic arrangements of NCX and CALX can diverge dynamic features of CBDs interdomain movements, thereby causing the opposite regulatory responses to Ca^2+^ (see below).

#### 4.2.2. Na^+^-Induced Inactivation: Where Does the Na^+^ Site Locate and How Does It Operate?

In general, two distinct mechanisms can alleviate the Na^+^-induced inactivation in mammalian NCXs: the first one refers to Ca^2+^ binding to CBD2 and the second one represents PIP_2_ interactions with a putative site nearby CBD2 [40,77,81,105,106,107,108,109,110,111,112]. A rise in the cytosolic Na^+^ rapidly increases the NCX-mediated ion currents caused by the Na^+^ interaction with the ion-transport sites; however, after reaching the peak values, the signal slowly decreases before reaching much lower steady-state levels (the I_1_-inactivation or Na^+^-induced inactivation). Even though the identity of the Na^+^ inactivation site remains puzzling, it is quite clear that Na^+^ does not interact with the Ca^2+^ sites at CBDs [120]. In contrast with NCX1 and NCX3, the Na^+^-induced inactivation was not detected for NCX2, thereby suggesting that the Ca^2+^ or ATP/PIP_2_-induced alleviation is irrelevant in the case of NCX2 [77,81,105,106,107,108,109,110,111,112]. Like NCX1, NCX3 also exhibits Na^+^-induced inactivation, although NCX3 lacks ATP/PIP_2_-induced alleviation of the Na^+^-induced inactivation [77,81,105,106,107,108,109,110,111,112].

**Figure 5:**
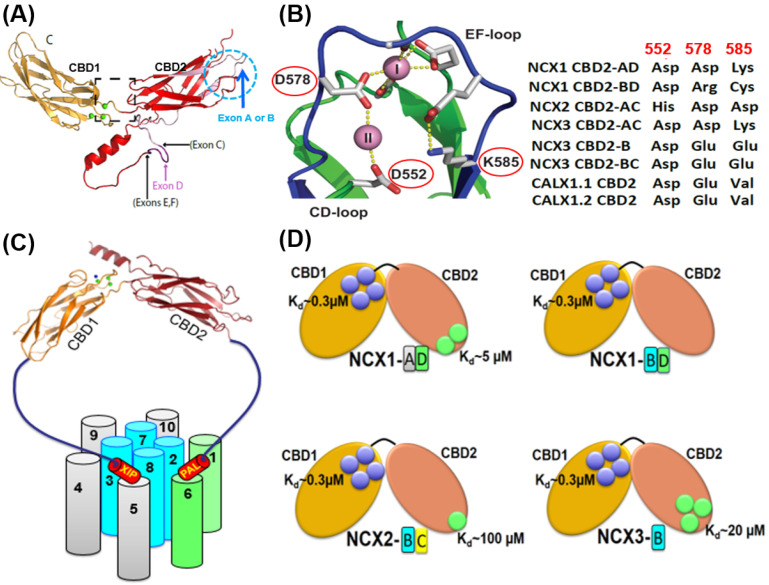
Structural basis of exon-dependent regulatory diversity in eukaryotic NCX variants. (**A**) Experimentally obtained NMR structures of CBD1 (2FWS), CBD2-AD (2FWU), and CBD2-BD (2KLT) of NCX1 were superimposed on the template of NCX1-CBD12 crystal structure (PBD 3US9) to demonstrate the structural positions of mutually exclusive (A or B) and cassette (CDEF) exons at CBD2. (**B**) The crystal structure of NCX1-CBD2-AD (PDB 2QVM) shows that K585 forms a salt bridge with D552 and E648 in the absence of Ca^2+^ (thereby stabilizing apo-CBD2), whereas in the presence of Ca^2+^, two Ca^2+^ binding sites (CaI and CaII) can be occupied by Ca^2+^. Different residues at the three key positions (522, 578, and 585) can predefine the number of Ca^2+^ binding sites at CBD2 in an exon-dependent manner. For example, in NCX1, exon B variants contain Arg (instead of Asp or Glu) and Cys (instead of Lys) at positions 578 and 585, respectively, which presents Ca^2+^ binding to CaI of NCX1-CBD2, thereby destabilizing the CBD2 folding. In contrast, the A-exon containing variants of NCX1 retain its structural integrity even in the absence of Ca^2+^ since K585 can form salt bridges with neighboring negatively charged residues (E516, D522, D578, and D578). In NCX2-CBD2 (which lacks splice variants), the substitution of D552 by histidine eliminates the CaII site while reducing the Ca^2+^ affinity at CaI. In NCX3, B-exon K585 is substituted by glutamate, generating three Ca^2+^ binding sites at CBD2. In contrast, the A-exon containing NCX3 variant has K585, which prevents Ca^2+^ binding to CBD2 (since K585 can form strong interactions with E516, D522, D578, and D578 in the absence of Ca^2+^). (**C**) Topological location of the regulatory CBD1 and CBD2 domains with regarding the autoinhibitory (XIP) and palmitoylation (PAL) domains. The ion-transporting helices (TM2, TM3, TM7, and TM8) are in blue. The sliding cluster (the TM1/TM6 bundle) is in green. The image of the regulatory two-domain tandem (CBD12) is presented according to the crystal structure of CBD12-NCX1.4 (PBD 3US9). (**D**) The Ca^2+^ binding sites of CBD1 (shown in blue circles) have a comparable affinity (K_d_ ≈ 0.3 µM) among NCX isoform/splice variants. In contrast, the K_d_ values of the Ca^2+^ binding sites of CBD2 (shown in green circles) vary from 5 µM to 100 µM. Thus, the exon-dependent structural variances control the number of Ca^2+^ binding sites (from zero to three) at CBD2 and the Ca^2+^ binding affinity.

It has been proposed that the Na^+^-inactivation site is located on the catenin-like domain at the N- and C-terminals of the cytosolic f-loop (5L6) [37,38]. In the absence of structural information, this proposal remains highly hypothetical. An alternative possibility is that the Na^+^ inactivation site is located at transport sites or nearby domains, where the Na^+^ binding to respective sites results in a slow accumulation of inactive species [77,116,117,118,119,120]. It was proposed that the allosteric Na^+^ binding to some unknown site somehow affects the domain–domain interactions between the auto-inhibitory XIP domain with neighboring domains, yielding a slow inactivation of NCX [169,174]. Notably, the kinetics and amplitude of Na^+^-dependent inactivation differ among the cardiac (ACDEF), brain (AD), and kidney (BC) variants of NCX1 [64,167,168,169,171], therefore suggesting that the splicing segment modulates the Na^+^-dependent inactivation. Notably, the NCX1 and NCX3 splice variants exhibit diverse capacities for Ca^2+^ dependent alleviation of Na^+^-induced inactivation since the mutually exclusive exons A and B predefine the number of Ca^2+^ sites at CBD2 by placing the Ca^2+^-coordinating residues at three critical locations (Figure 2A,B and Figure 5A,B).

#### 4.2.3. Multiple Proton Sensors May Contribute to the Proton-Dependent NCX Inactivation

Previous studies have shown that even mild acidification of the cytosolic pH in intact cardiomyocytes results in a dramatic shift in the [Ca^2+^]-dependent activation curve, so much higher concentrations of Ca^2+^ are required for NCX1.1 activation [120]. These observations, in conjunction with the Ca^2+^ binding assays to the isolated preparations of CBD1, CBD2, and CBD12 proteins, revealed that protons compete with Ca^2+^ for the occupation of the CBD sites. Thus, in contrast with Na^+^, protons and Ca^2+^ can cause opposite regulatory effects in full-size NCX1.1 by competing for the Ca^2+^ sites at the regulatory CBD domains. Even though this allosteric mode of proton-dependent inhibition of NCX1.1 can operate under altered physiological conditions (e.g., acidosis/ischemia), some other complementary mechanisms may contribute to the proton-dependent inhibition of NCX1.1, as well. For example, it was reported that two histidine residues, H124 and H165 (located on the short inter-helical loop at the cytosolic side), can significantly contribute to proton-dependent inhibition of NCX1.1 [175,176].

It has been assumed for many years that proton-dependent regulation of NCX does not involve protonation/deprotonation of ion-coordinating residues at the transport sites of NCX [7,8,26,63]. However, this postulation has been questioned in recent studies using the model system of NCX_Mj [71]. Notably, the pH titration curves of the Na^+^/Ca^2+^ exchange characteristically differ in a native NCX1.1 and NCX_Mj [71,82,98,177,178]. More specifically, the ion-coordinating carboxylates of NCX_Mj are deprotonated at pH > 4.5, thereby reaching the maximal rates of Na^+^/Ca^2+^ exchange already at pH 5.5 [71,82], thereby suggesting that the proton block mechanism is irrelevant for NCX_Mj. In contrast with NCX_Mj, the NCX1.1-mediated Na^+^/Ca^2+^ exchange rates increase from pH 5.5 to pH 10.5 [98,177,178]; Given the fact that three ion-coordinating residues (T50S, E213D, and D240N) differ between the mammalian NCXs and NCX_Mj (out of twelve), the relevant residues were replaced in NCX_Mj to test their effects on the pH-titration curve profiles. Strikingly, the T50S replacement nearly completely recapitulates the pH titration curves of NCX1.1-mediated Na^+^/Ca^2+^ exchange, meaning that T50S can account (at least partially) for the differences in the pH titration curve profiles observed between NCX_Mj and NCX1.1 [71]. The effect of T50S on the pH-dependent curves of ion exchange is fascinating from a structural standpoint since the appropriate structural arrangements may establish a basal mechanism for allosteric regulation through proton interactions with multiple allosteric domains (including CBDs, histidine sensors, and perhaps some others, as well). More specifically, the crystal structure of NCX_Mj [27,28], in conjunction with mutational studies [71,72], revealed that the backbone carbonyl of T50 coordinates Ca^2+^ (at S_Ca_). In contrast, the side chain of T50 can ligate either Na^+^ (at S_int_), or T50 can interact with N232 through hydrogen bonding. The S → T substitution at a matching T50 position in mammalian NCXs may cause an acidic shift in the pH-titration curve, as observed in NCX_Mj. Thus, the possibility is that the T50-matching serine residue in mammalian NCX can establish a basal condition for the proton-dependent inhibition of eukaryotic NCX at physiological pHs, which could be further amplified by proton-dependent allosteric regulation through CBDs, histidine sensors, and perhaps other regulatory modes, as well.

## 5. Structure-Dynamic Determinants of Regulatory Divergence in Eukaryotic NCXs

About 280 residues are directly involved in the folding of the CBD1 and CBD2 domains [39,40,41,42]. The X-ray and NMR structures of the CBD1, CBD2, and CBD12 domains reveal a β-immunoglobulin (Ig)-like folding, where two antiparallel β-sheets (with A-B-E and D-C-F-G strands) form a seven-strand β-sandwich motif [37,38,39,40,41,42]. The remarkable similarity between the folding structures of CBD1 and CBD2 is evident since the overlay of the CBD1 and CBD2 crystal structures display nearly identical folding with RMSD = 1.3 Å. In contrast, the Ca^2+^ binding sites in both CBDs reside at the C-terminal ends of distal loops [39,40,41,42]. Despite these structural similarities, CBD1 and CBD2 differ in the number of Ca^2+^ binding sites and coordination chemistry. However, the Ca^2+^ binding affinity at both CBDs is tightly controlled by splicing segments and might be of primary physiological significance [3,8,26]. In the CBD1 and CBD2 of NCX1, the Ca^2+^-coordinating residues are located at the AB, CD, and EF loops, although in addition to these three loops, the FG loop takes part in building the Ca^2+^-binding cluster at CBD2 [37,38,39,40,41,42]. Moreover, the contribution of the FG loop to Ca^2+^ coordination is controlled by the splicing segment, which determines the stoichiometry and affinity of Ca^2+^ at CBD2 [38,40,41,42] (Figure 5A–C).

### 5.1. Structure-Functional Specificities of High-Affinity Ca^2+^ Binding Sites at CBD1

Structural studies and Ca^2+^-binding assays revealed that the CBD1 of eukaryotic NCX and CALX orthologs, isoforms, and splice variants contain four Ca^2+^ binding sites (Ca1–Ca4). In contrast, the number of Ca^2+^ binding sites at CBD2 varies from zero to three (CaI–CaIII) due to the alternating splicing segment at CBD2 [39,40,41,42,116,117,118,119,120,121,122,123,124]. The four Ca^2+^ binding sites of CBD1 are assembled in a parallelogram-like configuration. In contrast, pol coordination of Ca^2+^ ions by the D500 and E451 residues allows one to concomitantly ligate two and three Ca^2+^ ions, respectively [39,41,42]. This structural organization of closely located Ca^2+^ binding sites (~4 Å) allows the Ca^2+^ binding to CBD1 with high cooperativity, which is essential for activation of NCX under physiologically relevant conditions when cytosolic [Ca^2+^] undergoes relatively small changes [52,55,119,120]. Notably, the C3 and C4 sites of CBD1 possess a high affinity for Ca^2+^ binding (K_d_ ≈ 0.2–0.5 µM) [119,170], thus representing a primary allosteric sensor for Ca^2+^-dependent activation of mammalian NCXs [116,117,118,119,120]. Based on the sequence similarities, the CBD1 folding might be very similar in NCX1, NCX2, and NCX3. Notably, the CBD1 crystal structure of CALX1.1 [41] shows striking similarities to the CBD1 structure of NCX1 [39,42]. The common structural features of CBD1 are remarkable since, in contrast with full-size NCXs (which undergo activation upon Ca^2+^ binding to CBD1), the Ca^2+^ binding to CBD1 of full-size CALX1.1 results in inhibition [167,168,169,171,172,173].

Although the K_d_ values of Ca^2+^ binding to the high-affinity sites of CBD12 are comparable among the cardiac (ACDEF), brain (AD), and kidney (BD) splice variants of NCX1, the Ca^2+^ dissociation rates from the high-affinity sites differ up to 200-fold [24,25,116,117,118,119,120,121,122,123,124]. More specifically, the stopped-flow measurements have identified slow rates (0.02–3 s^−1^) for the occluded Ca^2+^ dissociation from the high-affinity regulatory sites of CBD12 obtained from the different isoform/splice variants of NCX and CALX. These slow Ca^2+^ off-rates correlate remarkably with slow inactivation kinetics of full-size NCX variants, which were measured using patch-clamp techniques upon removal of the cytosolic Ca^2+^ [77,80,105,167,168,169,171]. Strikingly, the slow dissociation of regulatory Ca^2+^ can be observed only in the CBD12 constructs (the two-domain tandem) but not in the isolated CBD1 or CBD2 ones—this means that specific synergistic interactions between the two regulatory domains generate a slow dissociation of occluded Ca^2+^ [119,120,121,122,123,124]. The structure-based mutational analyses of CBD12-NCX1.4, CBD12-CALX1.1, and CBD12-CALX1.2 have shown that Ca^2+^ occlusion at the high-affinity C3 and C4 sites of CBD1 results in the Ca^2+^-dependent tethering of CBDs through a hydrogen-bonding network [41,42,118,119,120,121,122,123,124]. Thus, slow dissociation of occluded Ca^2+^ (due to the Ca^2+^ driven tethering of CBDs) can couple diverse regulatory phenotypes in NCX and CALX.

### 5.2. Varying Compositions of Exons Control the Affinity and Number of Ca^2+^ Sites at CBD2

In contrast with CBD1, the Ca^2+^ binding sites of CBD2 are ~5.5 Å apart, where K585 (a homolog to E454 in CBD1) forms a salt bridge with D552 and E648 (in the absence of Ca^2+^), thereby yielding a relatively more stable apo-CBD2 structure [40,42]. Cumulative data revealed that the mutually exclusive exons (A and B) control three positions (522, 578 and 585) in CBD2 that predefine the number of Ca^2+^ binding sites at CBD2 [37,38,39,40,41,42,116,117,118,119,120,121,122,123,124] (Figure 5B). The addition of cassette exons (CDEF) to the splicing segment shapes the Ca^2+^ binding affinities at both the CBD1 and CBD2 domains and modulates the Ca^2+^ dissociation rates from high-affinity regulatory sites to control NCX responses to cell-specific Ca^2+^ signaling/homeostasis [117,118,119,120,121,122,123,124]. Notably, in NCX1, the B-exon variants contain Arg (instead of Asp or Glu) and Cys (instead of Lys) at positions 578 and 585, respectively (Figure 5A,B); this prevents Ca^2+^ binding to CBD2 (e.g., in the kidney NCX1.3 variant). These structure-controlled arrangements significantly impact the regulatory capacity since the B-exon-dependent prevention of Ca^2+^ binding to CBD2 aborts the Ca^2+^-dependent alleviation of Na^+^-induced inactivation (Figure 1A,B). In the CBD2 of NCX2 (no splice variants), the replacement of D552 by histidine eliminates the CaII site while dramatically reducing the Ca^2+^ affinity at the CaI site (K_d_ ≈ 100 µM) [124]. However, in the absence of Na^+^-induced inactivation of NCX2, the effect of potential Ca^2+^ binding to CBD2 cannot be tested. In NCX3, B-exon replaces K585 with Glu to generate three Ca^2+^ binding sites at CBD2, whereas K585 in the A-exon of NCX3 prevents Ca^2+^ binding to CBD2 (since K585 interactions with E516, D522, D578, and D578 preclude Ca^2+^ binding) (Figure 5B).

Like mammalian NCXs, CALX1 also undergoes alternative splicing at CBD2, although the splicing segments of CALX1.1 and CALX1.2 are much shorter and differ from each other by only five residues [41,65]. Structural studies have shown that these five residues in CALX1-CBD2 are located within an FG-loop between the H1 α-helix and the β-strand, similar to the cassette exons’ (C, D, E, and F) positions that appear in mammalian NCXs [41,42]. Notably, the CBD2 domain of the CALX1 splice variants does not bind Ca^2+^.

### 5.3. The CBD1-CBD2 Linker and Dynamic Coupling of Ca^2+^-Dependent Tethering of CBDs

Comprehensive analysis of mutants using stopped-flow assays revealed that a short interdomain linker (501-HAGIFT-506) connecting the two CBDs is essential for structure-based regulatory coupling [42,116,117]. Notably, the interdomain CBD1-CBD2 linker is highly conserved among all known NCX and CALX variants. The CBD12 structural crystal structures of the NCX1.4, CALX1.1, and CALX1.2 underscore the crucial role of the CBD1-CBD2 linker in the Ca^2+^-dependent tethering of the CBD1 and CBD2 domains [41,42]. Genetically encoded elongation of the CBD1-CBD2 linker accelerates (up to 50-fold) the occluded Ca^2+^ off-rates and decreases the affinity of Ca^2+^ binding (up to 10-fold) at the high-affinity Ca3-Ca4 sites of CBD12, either in NCX or CALX [24,25,26,116,117,118,119,120,121,122,123,124]. Mutational analysis revealed that G503 is the only residue in the linker whose mutation abolishes the slow dissociation of occluded Ca^2+^ and alters the interdomain movements of CBDs [42,116]. Moreover, the crystal structures of NCX1-CBD12 and CALX-CBD12 indicate that the dihedral φ/ψ angles at position 503 are only allowed for the glycine residue [41,42] (any other residue at this position in the linker would result in a steric clash of protein folding). Functional analyses of full-size NCX and CALX have shown that mutations of either G503 in NCX1.1 or analogous G555 in CALX1.1 abort the Ca^2+^-dependent regulation of NCX ion currents [167,168,169,171]. Thus, the highly conserved CBD1-CBD2 linker controls the Ca^2+^-dependent interdomain tethering/coupling of CBDs in NCX and CALX.

### 5.4. The Structure of the Two-Domain Interface Predefines the Dynamic Coupling of CBDs

The discovery of the two-domain tandem (CBD12) crystal structures [41,42] provided a basis for elucidating the structure-dynamic determinants that predefine positive, negative, and no response to regulatory Ca^2+^ in NCX and CALX variants. The X-ray structures of the brain NCX1-CBD12-AD, CALX1.1-CBD12, and CALX1.2-CBD12 variants depict a relatively small contact area (~360 Å^2^) between the CBD1 and CBD2. Notably, the Ca^2+^ coordination chemistry is very similar in isolated CBD1, CBD2, and CBD12 domains, derived from either NCX or CALX orthologs. However, a few structural differences have a primary mechanistic significance [41,42], as specified below. Namely, E385 only coordinates Ca3 in isolated CBD1, whereas this residue contributes to Ca^2+^ ligation at the Ca2, Ca3, and Ca4 sites in the CBD12 of NCX1-AD, CALX1.1, or CALX1.2. The most important structural difference between the isolated CBD1 and CBD12 is that D499 forms bidentate coordination with Ca4 in CBD12 in contrast with monodentate coordination in isolated CBD1. The significance of these structural arrangements is that they may predefine the structural stability of Ca^2+^-dependent tethering of CBDs in NCX and CALX [3,5,117,118,119,120,121,122,123,124].

**Figure 6:**
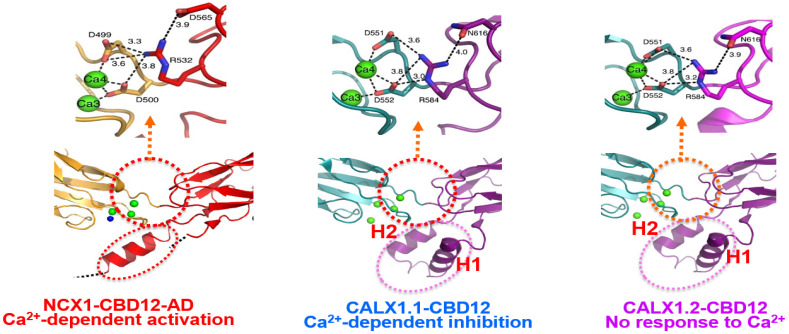
The interface structures of the two-domain CBD12 tandem in the NCX and CALX orthologs. Carton presentation of NCX1-CBD12-AD (PDB 3US9) and CALX1.1-CBD12 (PDB 3RB5) and CALX1.2 (PDB 3RB7) crystal structures depict highly conserved residues participating in the network of interdomain salt bridges. Green balls indicate the Ca^2+^ binding sites at the interface of NCX1-CBD12-AD, CALX1.1-CBD12, and CALX1.2-CBD12. The blue ball in NCX1-CBD12 refers to the Ca^2+^ binding site that cannot be resolved in the PDB 3US9 crystal structure, although the position of this Ca^2+^ binding site is nearly identical to the Ca^2+^ binding site of CALX1.1-CBD12 (PDB 3RB5) and CALX1.2 (PDB 3RB7) as revealed by these crystal structures. The Ca^2+^ occlusion occurs at the high-affinity binding sites (Ca3-Ca4) of CBD1 in all three proteins (shown in the red-dotted cycle). Although the interdomain salt-bridge structures are highly conserved among NCX and CALX variants, a striking difference between the NCX and CALX variants occurs in the structural organization of the FG-loop α-helix at CBD2. More specifically, the CALX1.1-CBD12 and CALX1.2-CBD12 variants have a two-headed (H1 and H2) short-helix structure, which situates nearly perpendicularly to the β-sheet plane of CBD2. In contrast, the α-helix of NCX1-CBD12 is longer, while adopting a straight configuration. These structural disparities in the α-helix folding (in conjunction with variations in exon composition) can predefine the dynamic features of CBDs movements and, thus, the regulatory specificities of a given ortholog/isoform/splice variant. Thus, the structure-encoded dynamic distinctions of relevant structural elements may predefine (at least partially) the characteristic responses of full-size NCX and CALX variants to regulatory Ca^2+^.

In general, the crystal structures of NCX1-CBD12 and CALX1-CBD12 revealed three regions (assigned as the hydrophilic, hydrophobic, and loop/α-helix arrays), which involve over 20 residues [41,42,172,173]. The hydrophilic region includes a pivotal electrostatic network centered at R532 in CBD2, where R532 forms a bifurcated network of salt bridges with D499 and D500 in CBD1 and D565 in CBD2 once Ca3-Ca4 become occupied by Ca^2+^ [42]. Notably, this Ca^2+^-related tethering (through D499 and D500) contributes to two Ca^2+^ coordination at Ca3-Ca4 while concomitantly stabilizing the CBD interface (Figure 6). Thus, this highly conserved network of salt bridges acts as a major linchpin that holds the two CBDs together upon Ca^2+^ occlusion at the two-domain interface. The Ca^2+^-dependent rigidification of the two-domain interface is further supported by SAXS and HDX-MS analyses of isolated CBD12 variants [42,118,119,120,121,122,123,124]. Thus, the coupling of Ca^2+^ occlusion with CBD tethering represents a unifying mechanism for interdomain coupling, where the structure-dynamic stability (rigidity) of the interdomain realm can be gradually modulated by varying composition of exons [3,8,25,26,119,120,121,122,123,124].

The hydrophobic region at the two-domain interface contains residues that locate on the Ca^2+^-binding EF loop of CBD1, the interdomain linker, and the FG loop of CBD2. The crucial point is that F450 interacts with H501, I628, A629, M631, and G632 (through van der Waals interactions) to form a tightly packed hydrophobic core [42]. Notably, these residues are inaccessible to the bulk phase, so even minute structural changes within this tightly packed hydrophobic core can significantly affect the dynamic features of interdomain motions. For example, the Ca^2+^-dependent interaction of F450 with H501 may limit the flexibility of the interdomain linker. Moreover, neighboring interactions between the CD and EF loops of CBD1 and the FG-loop of CBD2 (including the splicing segment) can modulate the Ca^2+^ binding affinity to the Ca3-Ca4 sites of CBD1. Since the FG-loop of CBD2 is unstructured (except for a short α-helix region at the C-terminus of the FG-loop), it is reasonable to assume that the side chains of a canonical α-helix impact the structure-dynamic features of the two-domain interface [42]. Notably, the FG-loop of CALX1-CBD12 forms a two-headed short helix structure (H1 and H2), upright to the β-sheets [41]. This structural organization of CALX-CBD12 is strikingly different from the matching helix structure of NCX1-CBD12-AD [42] (Figure 6).

The α-helix region (belonging to the FG-loop of CBD2) is very close to the CBD1-CBD2 linker and the Ca3-Ca4 sites of CBD1 (either in NCX or CALX), thereby suggesting that the relevant interactions may affect the Ca^2+^ access to high-affinity binding sites of CBD1. The two-headed short helices (H1 and H2) of CALX can more effectively stabilize the interdomain linker and CBD2 folding as compared with mammalian NCX variants, having a straight and longer α-helix at the matching position. These “minute” differences between NCX1 and CALX1 in the structural organization of the FG-loop α-helix can differently affect the rigidity of the interdomain linker and CBD2 folding [119,120,121,122,123,124].

### 5.5. Dynamic Features Might Predefine the Opposing Responses of NCX and CALX to Regulatory Ca^2+^

Based on the crystal structures of Ca^2+^-bound CALX1.1-CBD12 and CALX1.2-CBD12, it was suggested that slight differences in the interdomain angle (~8º) between the CBD1 and CBD2 domains determine different responses to regulatory Ca^2+^ in full-size CALX1.1 and CALX1.2 variants [41]. However, the crystal structure of NCX1-CBD12 demonstrated that the interdomain angle of Ca^2+^-bound CBD12 is nearly identical for NCX1-CBD12-AD (117.4°) and CALX1.1-CBD12 (117.7°), which means that the fixed-angle alignment of CBDs cannot account for Ca^2+^-dependent activation (NCXs) and inhibition (CALX1.1) [42]. Cumulative data obtained by NMR [125,179,180,181], SAXS [117,118,119,120,121], and HDX-MS [120,121,122,123,124] reveal a common model for Ca^2+^-dependent activation of mammalian NCXs. According to this unifying mechanism, the Ca^2+^-dependent activation of mammalian NCXs involves the Ca^2+^-dependent tethering of two CBDs (due to the Ca^2+^ occlusion), which restricts the interdomain movements of CBDs to keep mammalian NCX active. Subsequently, a slow dissociation of occluded Ca^2+^ leads to NCX inactivation, where the time scale of NCX activation depends on the kinetics of the occluded Ca^2+^ dissociation. Even though the interdomain network of R532, D499, and D500 residues is a common structural module for the Ca^2+^ occlusion and subsequent tethering of CBDs either in NCX or CALX (Figure 6), the structure-dynamic stability of the two-domain tethering may predefine the regulatory outcomes of the Ca^2+^-dependent regulation in distinct variants. The emerging working hypothesis is that the structure-dynamic stability of the two-domain tethering might differ among NCX and CALX variants since effects of the two-domain interface, the CBD1-CBD2 linker, and varying compositions of exons characteristically differ among NCX and CALX variants [42,119,120,121,122,123,124]. For example, it is reasonable to assume that the two-domain tethering is less stable in CALX than in NCX since the off-rates of the occluded Ca^2+^ are much faster in CALXs (3–12 s^−1^) than in NCXs (0.03–0.5 s^−1^) [42,117,118,119,120,121,122,123,124]. How these dynamic differences in the Ca^2+^ tethering could be related to translational and rotational movements of CBDs, remains to be resolved (see below).

### 5.6. Population Shift Mechanism Can Account for Opposite Responses to Regulatory Ca^2+^

The analyses of CALX1.1-CBD12, CALX1.2-CBD12, NCX1-CBD12, NCX2-CBD12, and NCX3-CBD12 by SAXS have shown that the occupation of the Ca3-Ca4 sites by Ca^2+^ shifts the fractional distribution of conformational states toward a more constrained population [42,117,118,119,120,121]. These findings revealed that the Ca^2+^ binding rigidifies the interdomain movements of CBDs, where the average distance between the CBD1 and CBD2 (as well as their alignment) remains unaffected. These findings are consistent with the population shift mechanism, according to which the ligand (Ca^2+^) binding to a given protein does not generate new conformational states; instead, the ion binding shifts a predominant population of unstable conformational states to a new population of more stable conformational states [118,182,183,184,185,186,187,188,189,190]. Thus, upon Ca^2+^ binding, the fraction of more rigid (constrained) conformational states becomes dominant at dynamic equilibrium. The unique feature of the population shift mechanism is that it avoids large conformational transitions requiring sizable free energy changes [182,185,190]. The advantage of the population shift mechanism versus the induced fit mechanism (an alternative mechanism to the population shift mechanism) is that the induced fit can operate under one of two scenarios: when ligand (ion) concentrations are very high or when the protein has a very high affinity for ligand binding [115,116,117,118,182,183,184,185]. Neither of these conditions fits the Ca^2+^-dependent regulation of NCXs since the quick and effective response of tissue-specific NCX variants is required to match the Ca^2+^-dependent events that occur from the millisecond time range to minutes and hours [182,183,184,185,186,189].

Consistent with the population shift mechanism, NMR analysis revealed that Ca^2+^ binding to the Ca3–Ca4 sites of NCX1-CBD12 or CALX-CBD12 restricts the linkers’ flexibility and interdomain movements of CBDs [125,179,180,181]. These data are consistent with the SAXS and HDX-MS findings, revealing that Ca^2+^ occlusion at Ca3-Ca4 of NCX and CALX rigidifies the backbone dynamics of the two-domain interface, which is coupled with CBD tethering through Ca^2+^ occlusion [41,42,116,117,118,119,120,121,122,123,124]. Interestingly, the global structural parameters of CBDs (e.g., the maximal interdomain distance and the radius of gyration) are similar in the apo- and the Ca^2+^-bound forms in all tested variants (the NCX1.4-CBD12, CALX1.1-CBD12, and CALX 1.2-CBD12 variants), although the Ca^2+^ binding narrows and shifts the population of conformational states under a dynamic equilibrium [42,118]. Although NCX1.4-CBD12, CALX1.1-CBD12, and CALX 1.2-CBD12 show striking similarities in Ca^2+^-dependent tethering [41,42], the stability of this network is diverged by the two-domain interphase and varying exon compositions [116,117,118,119,120,121,122,123,124].

### 5.7. Allosteric Signal Transfer from CBD1 to CBD2 May Modulate the TM1/TM6 Sliding

**Figure 7:**
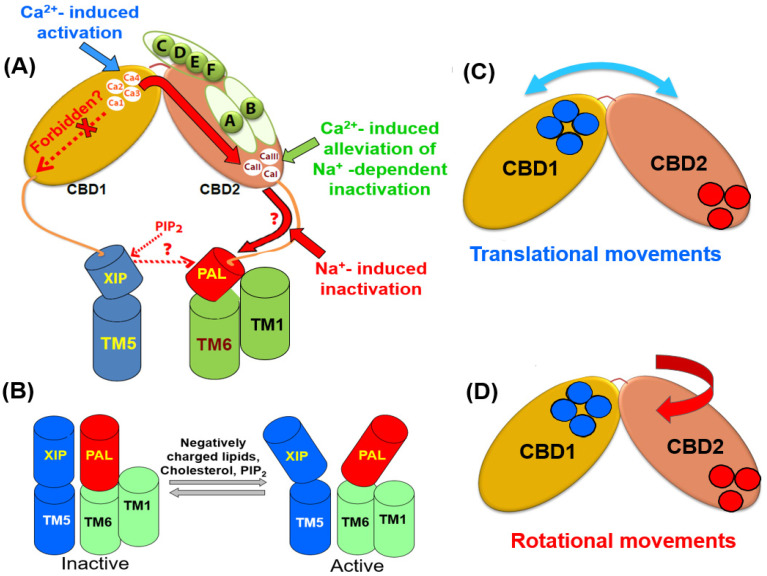
Structural elements that contribute to the propagation and integration of allosteric signals. (**A**) The occupation of the Ca3-Ca4 sites by Ca^2+^ rigidifies the backbone dynamics at the two-domain interface of CBDs. The allosteric signal propagation from the C-terminal tip of CBD1 to the C-terminal end of CBD2 (shown in a solid red arrow) is under the control of the splicing segment at CBD2 and the two-domain interface. (**B**) The positively charged XIP (autoinhibitory) domain can anchor the negatively charged helix (PAL) at the palmitoylation site. Since the PAL domain links to TM6, the XIP/PAL unit may affect the sliding of the TM1/TM6 bundle, thereby modulating the transport rates. The interactions of inhibitory (e.g., Na^+^) and activating (e.g., PIP_2_) ligands with respective sites (located of the XIP/PAL unit) can shift a steady-state equilibrium between the active and inactive states. Blue and red cylinders represent the XIP and PAL domains, respectively. The interdomain movements of the CBD1 and CBD2 domains might involve translational (**C**) and rotational (**D**) movements. Specific structural elements (e.g., the two-domain interface, XIP/PAL unit, and varying exon compositions) may characteristically shape the outcomes of regulatory specificity in a given NCX variant by controlling a relationship between the translational and rotational movements. The blue and red cycles represent the Ca^2+^ binding sites of CBD1 and CBD2, respectively.

The HDX-MS analyses of CBD12 preparations revealed that the Ca^2+^-dependent tethering of CBDs rigidifies the CBD interface in NCX and CALX [42,120,121,122,123,124]. However, the strength and spread of the Ca^2+^-dependent rigidification characteristically differs in NCX1-CBD12 (AD), NCX2-CBD12, NCX3-CBD12 (BC), and CALX1.1. These differences in the backbone rigidification are especially prominent when comparing NCX and CALX variants. In NCX1-CBD12-AD, the Ca^2+^ binding to CBD1 rigidifies the backbone from the Ca3–Ca4 sites of CBD1 up to the C-terminal tip of CBD2 (Figure 7). This allosteric pathway covers a distance of ~50 Å, which also embraces the CBD2 α-helix, including the splicing segment. Notably, the mutation of F450 (a central residue with the hydrophobic core of the two-domain interface) aborts the propagation of the Ca^2+^-dependent rigidification of CBD2. Thus, the F450-dependent stabilization of the CBD1-CBD2 linker is essential for transferring the allosteric massage from CBD1 to CBD2. In CALX1.1-CBD12 the propagation of the Ca^2+^-dependent rigidification of the backbone also begins from the Ca3-Ca4 sites. However, the rigidification occurs in a very short distance that stops at the CBD2 α-helix [122,123,124]. The NMR [125,179,180,181] analyses [125,179,180,181] provided consistent and complementary information on the relevant issues. According to a fundamental paradigm, two domains connected through a short interdomain linker (as occurs in CBD12) might undergo transitional and rotational movements (Figure 7C,D). The output of transitional and rotational movements could be characteristically different among NCX and CALX variants. In mammalian NCXs, the allosteric signal propagates from the C-terminal tip of CBD1 to the C-terminal CBD2 tip. The splicing segment can specifically shape a relationship between the translational and rotational movements in a given isoform/splice variant, thereby yielding distinct regulatory profiles. In CALX, the unstable tethering of CBDs and folding stability of CBD2 modulate the translational and rotational movements in such a way that this leads either to inhibition or no response to regulatory Ca^2+^. The resolution of the underlying mechanisms is essential since the C-terminal of CBD2 links to TM6, meaning that the different allosteric pathways may affect the sliding of the TM1/TM6 bundle (Figure 7A,B). These structural arrangements may provide a basis for a cross-talk between the CBDs and transport domains either in NCX or CALX proteins. The analyses of nanodisc-reconstituted NCX and CALX using the Cryo-EM and HDX-MS techniques may provide crucial information on the positive, negative, and no responses to Ca^2+^.

### 5.8. Mutually Exclusive and Cassette Exons Operate through Different Regulatory Modules

Conceptually, the splicing segments of proteins contain intrinsically disordered regions, which avoids the formation of stable tertiary structures [185,186,187]. Instead, the splicing segments can embrace more stable conformational states upon ligand binding, which allows dynamic functional transitions in a given protein [186,187,188,189,190,191]. Consistent with this general concept, the Ca^2+^ binding to CBDs results in a population shift of numerous pre-existing conformational states through incremental (low energy) conformational transitions. The mutually exclusive exons, A and B, are located at the C-terminus tip of CBD2 (on the loops that are directly involved in Ca^2+^ coordination) [37,38,39,40,41,42]. Exons A and B play opposite roles in NCX1 and NCX3 [3,38,116,117,118,119,120,121,122,123,124]. In NCX1, exon B increases the Ca^2+^ affinity at the Ca3-Ca4 sites of CBD1 (while decelerating the off-rates of occluded Ca^2+^ dissociation from Ca3-Ca4), whereas exon B structurally prevents Ca^2+^ binding to CBD2 (Figure 5A–C). In NCX1, exon A forms two Ca^2+^ binding sites at CBD2, while decreasing Ca^2+^ affinity at CBD1. The roles of exons A and B exon become inverted in NCX3 as compared with NCX1—namely, in NCX3, exon A prevents Ca^2+^ binding to CBD2 while increasing the Ca^2+^ binding affinity at CBD1 (Figure 5B,C). In NCX3, exon B generates three Ca^2+^ sites at CBD2 (due to K585 replacement by glutamate, which also decreases the Ca^2+^ affinity at CBD1 (106–110). Thus, the mutually exclusive exons (A and B) not only condition the Ca^2+^ binding stoichiometry to CBD2 but also shape the Ca^2+^ affinity to CBD1 and CBD2 domains [117,120,121,122].

The cassette exons (C, D, E, and F) are located at the unfolded segment of the FG-loop while being in proximity with the high-affinity sites (Ca3-Ca4) of CBD1 and the interdomain CBD1-CBD2 linker [37,38,39,40,41,42] (Figure 5A). As a complementary unit for modulating the dynamic features of interdomain coupling, the cassette exons (C, D, E, and F) can effectively rigidify the interdomain linker thereby stabilizing the CBD2 folding [3,117,118,119,120,121,122,123,124]. Notably, the gradual additions of the cassette exons (C, D, E, and F) to exon A incrementally enhances the affinity of the Ca^2+^ binding sites at CBD1 as well as slows down the dissociation of occluded Ca^2+^ from the Ca3-Ca4 sites [119,120,121,122,123]. Notably, the gradual additions of the cassette exons compensate for the destabilizing effect caused by exon A on the high-affinity Ca^2+^ binding sites of CBD1. Most probably, the exon-dependent rigidification of the CBD2 BC-loop and interdomain linker (upon Ca^2+^ binding to the Ca3-Ca4 sites of CBD1) reduces the translational movement of CBD12. In contrast, the exon-dependent rigidification of the neighboring β-strands in CBD2 might restrict the rotational motion of CBD2. Besides the exons’ compositions, the intrinsic folding energy of CBD2 may affect the integration of transitional and rotational movements. For example, the intrinsic structure of CBD2 might be more rigid in CALX than in NCXs, which may limit the rotational movements of CBD2, thereby causing a negative or no response to Ca^2+^ in CALX1.1 or CALX1.2, respectively.

## 6. Lipids Modulate Mammalian NCXs through Unknown Mechanisms

Negatively charged phospholipids [192,193], anionic amphiphiles [81,109,110,111,112,194,195], fatty acids [196], phosphatidyl serine, cholesterol [100,101], and long-chain acyl CoA modulate mammalian NCX activity through unknown molecular mechanisms [197]. In most cases, lipids activate NCX activity, although some lipids cause inhibition [6,196]. In general, lipids oppose Na^+^-dependent inactivation [100,101,113,197,198,199]. It was proposed that the PIP_2_ binding to XIP (auto-inhibitory domain) activates NCX1.1, whereas the Na^+^-dependent release of PIP_2_ from XIP inhibits NCX [198]. An emerging working hypothesis is that without PIP_2_, a positively charged XIP helix (located on the TM5) anchors a negatively charged helix (situated on the CBD2-TM6 segment). According to this proposal, inhibitory (e.g., Na^+^) and activating (e.g., PIP_2_) ligands can shift between the steady-state equilibrium between the active and inactive states, thereby characteristically shaping the outcomes of regulatory effects in distinct isoform/splice variants [106,174,200] (Figure 7). The structure-functional mechanisms underlying the lipid interactions with NCXs may operate similarly to other ion-transporting proteins [102,201].

Another factor that may affect lipid–protein interactions is the varying composition of exons in the alternative splicing segment. For example, fatty acids more effectively inhibit NCX1.3 (BD), expressed in smooth muscle and kidney, than in the cardiac NCX1.1 (ACDEF) variant [103]. These differences in lipid-dependent inhibition could be associated with structure-dynamic and functional variances predefined by the existence of mutually exclusive exons A and B (see above). The potential contributions of cassette exons to the modulation of lipid–protein interactions are of general interest, since the underlying mechanisms may contribute to the stabilizations of the CBD1–CBD2 linker, which in turn controls the dynamic coupling of CBDs (see above Section 5.8).

In contrast with mammalian NCXs, prokaryotic NCX_Mj is insensitive to varying compositions of lipids [67]. Since negatively charged lipids and cholesterol activate the NCX1.1-mediated transport rates up to 10-fold [100,101], it is evident that lipid–protein interactions can contribute (at least partially) to the 10^4^-fold differences in the transport rates observed between NCX1.1 and NCX_Mj [55,56,57,58,59,60,67]. The lack of lipid-dependent modulation in NCX_Mj can be explained by the intrinsic stability (structural rigidity) of NCX_Mj as a thermophilic protein, although one must consider alternative possibilities, as well. At this end, it is unclear whether the lipid-dependent effects occur in other prokaryotic NCXs and, if so, how the lipid–protein interaction impacts ion-transport features in phylogenetically distant NCXs. More dedicated and systematic research is required to investigate lipid–protein interactions in prokaryotic and eukaryotic NCX variants.

## 7. Palmitoylation of Mammalian NCX: A Coupling Unit for Functional Integration?

Different post-translational modifications occur in NCX1, NCX2, and NCX3 isoforms, including glycosylation, S-palmitoylation, and Ca^2+^-dependent cleavage by proteases (e.g., by μ-calpain) among many others (for review see ref. [6]). Besides the S-palmitoylation [154,155,156,157,158], post-translational mechanisms underlying the NCX modification were not investigated systematically; thus, a piece of valuable information on the relevant issues is quite limited, unfortunately. The post-translational S-palmitoylation of mammalian NCXs seems interesting not only in terms of NCX insertion into the membrane and cellular trafficking but also from the structure-based modulation and integration of regulatory modes. Due to these reasons, only the S-palmitoylation mechanism is discussed below, while shortly summarizing the recently discovered molecular and cellular mechanisms and future perspectives [156,157,158,200].

Palmitoylation is a covalent addition of C16 fatty acids to protein cysteines via S-acylation, which controls the protein’s trafficking and insertion into the membrane while contributing to cellular signaling [159,160,161,162]. Recent studies revealed that the single palmitoylation site (C739) of NCX1 locates on the short α-helix (residues 740–756), where the enzymes performing the S-acylation of C739 can selectively recognize the abundant α-helix [156]. Although the NCX2 and NCX3 isoforms contain a highly conserved palmitoylation α-helix, two cysteine residues appear at the N- and C-terminal ends of the 740–756 helix (analogous to positions 739 and 757 in NCX1) [156,157,158]. The emerging working hypothesis is that selective palmitoylation of two cysteine residues can differently modulate NCX1, NCX2, and NCX3 isoforms [154,155,156,157]. Notably, the palmitoylation α-helix (residues 740–756) locates between the CBD2 and TM6. Therefore, the possibility is that the palmitoylation domain can affect the TM1/TM6 sliding and thus, the transport rates (Figure 7A,B). The palmitoylation α-helix residues may condition the interactions of negatively charged α-helix (residues 711–736) with positively charged autoinhibitory XIP domain (residues 255–275) (Figure 7A,B), which may predefine the status of inactive and inactive states. These structure-based domain–domain interactions could be essential for diverging and integrating distinct modulatory signals. The possibility is that structure-based hydrophobic interactions of the S-acyl moiety with membrane lipids and regulatory ligands (e.g., PIP_2_) modulate dynamic features of the TM1/TM6 movements, where the associated altering in the OF/IF swapping limits the transport rates. Future experiments using the advanced approaches of Cryo-EM and HDX-MS techniques may address these issues by examining the nanodisc-reconstituted full-size variants of mammalian NCXs.

## 8. Conclusions and Perspectives

During the last decade, remarkable progress has been achieved toward the understanding of structure-dynamic mechanisms underlying the ion transport and regulation of NCX orthologs/isoforms/splice variants. This was achieved by applying especially suited multidisciplinary approaches for systematic analyses of the archaeal (NCX_Mj), invertebrate (CALX1–2), and mammalian (NCX1–3) sodium–calcium exchanger proteins (as summarized in Section 2, Section 3, Section 4, Section 5, Section 6 and Section 7, above). The breakthrough discovery of the archaeal NCX_Mj crystal structure provided new opportunities for structure-based studies of ion transport mechanisms shared by eukaryotic and prokaryotic NCXs. These studies put forward a structure-functional model describing how the ion interactions with respective binding sites (owing diverse ion selectivity) induce an alternating exposure (access) of the ion-binding pocket at the opposite sides of the membrane. Despite this progress, the structure-dynamic determinants underlying the kinetic variances among the NCX variants remain unresolved. It is essential to resolve the structure-functional and dynamic features of ion-induced swapping of the OF and IF states as related to the alternating access mechanism. Future analyses of nanodisc-reconstituted NCX_Mj, using the advanced approaches of 19F-NMR and HDX-MS), may provide breakthrough information on the ion-induced alternating access transitions in NCX and similar proteins.

The high-resolution crystal structures of NCX_Mj represent an excellent structure-dynamic model for studying a common mechanism of ion transport in NCX variants. However, NCX_Mj cannot serve as a model system for elucidating the allosteric interactions between the regulatory and transport domains, since NCX_Mj lacks the regulatory domains. The crystal structures of isolated regulatory domains (CBD1, CBD2, and CBD12), derived from eukaryotic NCXs and their analyses using SAXS, FRET, and HDX-MS, provided very useful information on the structure-dynamic basis of regulatory divergence in eukaryotic NCXs. Despite this progress, the isolated regulatory domains cannot be explored for studying the remote allosteric interactions between the regulatory and ion-transporting domains. Thus, the emerging goal is to explore new experimental systems and approaches to elucidate how the regulatory massages become diverged, transferred, and integrated in tissue-specific NCX variants. This underscores an urgent need for the discovery of the full-size structure of eukaryotic NCX. Most probably, this will be achieved using the advanced techniques of Cryo-EM. In conjunction with the full-size structure of mammalian NCX, the advanced approaches of the CRISPR/Cas9 techniques can be applied for elucidating the function and regulation of tissue-specific NCX variants under physiological and pathophysiological conditions. Once the full-size structure of mammalian NCX becomes available, this can provide new opportunities for structure-based devising of drug-like ligands for selective inhibition or activation of tissue-specific NCX isoform/splice variants. In the long-term run, this may identify drug candidates, which could be considered for focused clinical trials.
